# Transcriptome analysis reveals gender-specific differences in overall metabolic response of male and female patients in lung adenocarcinoma

**DOI:** 10.1371/journal.pone.0230796

**Published:** 2020-04-01

**Authors:** Ya Li, Cheng-Lu He, Wen-Xing Li, Rui-Xian Zhang, Yong Duan

**Affiliations:** 1 Yunnan Key Laboratory of Laboratory Medicine, Kunming, Yunnan, China; 2 Yunnan Institute of Laboratory Diagnosis, Kunming, Yunnan, China; 3 Department of Clinical Laboratory, The First Affiliated Hospital of Kunming Medical University, Kunming, Yunnan, China; 4 Key Laboratory of Animal Models and Human Disease Mechanisms, Kunming Institute of Zoology, Chinese Academy of Sciences, Kunming, Yunnan, China; 5 Kunming College of Life Science, University of Chinese Academy of Sciences, Kunming, Yunnan, China; 6 Yunnan Center for Disease Control and Prevention, Kunming, Yunnan, China; West Virginia University, UNITED STATES

## Abstract

**Background:**

Evidence from multiple studies suggests metabolic abnormalities play an important role in lung cancer. Lung adenocarcinoma (LUAD) is the most common subtype of lung cancer. The present study aimed to explore differences in the global metabolic response between male and female patients in LUAD and to identify the metabolic genes associated with lung cancer susceptibility.

**Methods:**

Transcriptome and clinical LUAD data were acquired from The Cancer Genome Atlas (TCGA) database. Information on metabolic genes and metabolic subsystems were collected from the Recon3D human metabolic model. Two validation datasets (GSE68465 and GSE72094) were downloaded from the Gene Expression Omnibus (GEO) database. Differential expression analysis, gene set enrichment analysis and protein-protein interaction networks were used to identified key metabolic pathways and genes. Functional experiments were used to verify the effects of genes on proliferation, migration, and invasion in lung cancer cells in vitro.

**Results:**

Samples of tumors and adjacent non-tumor tissue from both male and female patients exhibited distinct global patterns of gene expression. In addition, we found large differences in methionine and cysteine metabolism, pyruvate metabolism, cholesterol metabolism, nicotinamide adenine dinucleotide (NAD) metabolism, and nuclear transport between male and female LUAD patients. We identified 34 metabolic genes associated with lung cancer susceptibility in males and 15 in females. Most of the metabolic cancer-susceptibility genes had high prediction accuracy for lung cancer (AUC > 0.9). Furthermore, both bioinformatics analysis and experimental results showed that TAOK2 was down-regulated and ASAH1 was up-regulated in male tumor tissue and female tumor tissue in LUAD. Functional experiments showed that inhibiting ASAH1 suppressed the proliferation, migration, and invasion of lung cancer cells.

**Conclusions:**

Metabolic cancer-susceptibility genes may be used alone or in combination as diagnostic markers for LUAD. Further studies are required to elucidate the functions of these genes in LUAD.

## Introduction

Lung cancer is one of the most common malignancies worldwide, and in China, the incidence of lung cancer is highest among all cancers [1, 2]. Non-small cell lung cancer (NSCLC) accounts for ~80% of all lung cancer cases and lung adenocarcinoma (LUAD) is the most common subtype. Altered metabolic processes are now widely recognized hallmarks of cancer cells, and involve the complex rearrangement of metabolic and energy producing networks to support the high proliferation rates of tumor cells and their unique metabolic demands [3]. Multiple studies have correlated cellular metabolic characteristics with degree of malignancy, which could potentially be used to predict of the prognosis of patients with NSCLC based on positron emission tomography/computed tomography (PET/CT) scans [4–6].

To date, a number of hub genes and metabolic pathways have been associated with lung cancer, and this valuable information may eventually reveal the mechanisms involved in the pathogenesis of lung cancer, leading to improved clinical treatments. Evidence shows abnormal glucose metabolism and fatty acid metabolism are directly related to LUAD [7, 8]. The glycolytic enzyme pyruvate kinase M (PKM) has two isoforms: PKM1 and PKM2. Several studies reported that high PKM1 expression intrinsically activates glucose metabolism and boosts tumor cell growth [7]. Another recent study found that higher expression of nuclear factor erythroid 2-related factor 2 (NRF2)-regulated metabolic gene signature (NRMGS) predicted poor overall survival in eight independent NSCLC cohorts [9]. Furthermore, over-expression of bone morphogenetic protein 4 (BMP4) has been correlated with acquired drug resistance and fatty acid metabolism in NSCLC cells with EGFR mutations [10]. Another study suggested altered glucose transporter 1 (GLUT1)-mediated glucose metabolism as a potential approach for treating NSCLCs resistant to EGFR inhibitors [11]. In recent years, the incidence of lung cancer has decreased among men but increased in women, mainly owing to changes in smoking patterns among the sexes. However, studies have shown that lung cancer is caused not only by cigarette smoke, but that other genetic and environmental factors may be at play [12]. Therefore, studies on the mechanisms involved in the development of lung cancer, as well as potential new treatments, should consider gender.

The establishment of human metabolic models has provided a considerable amount of data as well as systematic analytical methods for research on human metabolic disease [13, 14]. However, the role of metabolism in LUAD has not been extensively studied. To the best of our knowledge, no previous study has compared the expression profiles of metabolic genes in males and females with LUAD. In the present study, we obtained information from the Recon3D human metabolic model on all human metabolic genes and metabolic subsystems [14]. In addition, we acquired the LUAD transcriptome and clinical data from publicly available databases (Genomic Data Commons Data Portal, https://portal.gdc.cancer.gov/) with the aim of exploring similarities and differences in metabolic gene expression and metabolic subsystems between male and female LUAD patients. Finally, we screened the hub metabolic genes to further understand the link between metabolic genes and patient prognoses.

## Materials & methods

### Lung adenocarcinoma data collection

The LUAD transcriptomes and clinical data were downloaded from the Genomic Data Commons (GDC) Data Portal (https://portal.gdc.cancer.gov/). The complete transcriptome and clinical data for 513 tumor samples and 57 adjacent non-tumor samples were obtained, including 237 tumor samples and 23 adjacent non-tumor samples in males and 276 tumor samples and 34 adjacent non-tumor samples in females. We generated RNA sequencing (RNA-seq) data from all samples using the Illumina HiSeq 2000 platform (version 2) and gene expression based on the RNA-seq data were normalized by the fragments per kilobase of exon per million reads mapped (FPKM) method. To reduce background noise and ensure reliable detection, we selected only genes with a 90% log2 (FPKM) value greater than 0.1 and obtained 13782 unique genes. To evaluate the results our analysis, we downloaded two lung cancer microarray datasets from the Gene Expression Omnibus (GEO) at the National Center for Biotechnology Information (NCBI; http://ncbi.nlm.nih.gov/geo) as a comparison: the GSE68465 dataset containing 223 male patients, 220 female patients, and 19 controls without gender information and the GSE72094 dataset containing 202 male patients, 240 female patients, but no controls.

### Differential metabolic genes analysis

Human metabolic genes were extracted from the Recon 3D human metabolism model [14]. The model contains 3288 unique genes that belong to 110 metabolic subsystems. We mapped 2194 metabolic genes and 104 metabolic subsystems from our LUAD data. Differential metabolic gene analyses was performed using R Statistical Software (version 3.4.1; Foundation for Statistical Computing, Vienna, Austria, https://www.r-project.org/). The empirical Bayes algorithm of the limma package (version 3.30.13) [15] in R was used to detect genes differentially expressed between samples of tumor tissue and adjacent non-tumor tissue. Logarithmic transformation (log2) of all gene expression values was performed for each gene and fold change (log2(FC)) was calculated as the mean expression value in tumor samples minus the mean expression value in adjacent non-tumor samples. Genes were considered upregulated if log2(FC) ≥ 1 and false discovery rate (FDR) adjusted P value ≤ 0.05. Genes were considered downregulated if log2(FC) ≤ -1 and FDR-P value ≤ 0.05. Finally, we performed a differential expression analysis between tumors from male and female patients.

### Metabolic subsystem enrichment analysis

We used the javaGSEA desktop application in R (version 3.0; http://software.broadinstitute.org/gsea/) [16] to perform gene set enrichment analysis (GSEA) of the mapped metabolic subsystems in male tumor vs. male adjacent, female tumor vs. female adjacent, and male tumor vs. female tumor. Gene sets with less than 15 genes or more than 500 genes were excluded. The t-statistic mean of genes in each metabolic subsystem were then computed using a permutation test with 1000 replications. Subsystems with normalized enrichment scores (NESs) > 0 were considered upregulated and subsystems with NESs < 0 were considered down-regulated. Statistical significance was identified as P ≤ 0.05.

### Comparison of clinical variables and prognosis analysis of deregulated metabolic genes

In the clinical data analysis, continuous variables (e.g., age) were presented as mean ± one standard deviation (SD) and categorical variables (e.g., race, smoking history, tumor status and stage) were expressed as numbers (percentages). We used Student's t-tests to compare the differences in continuous variables between male and female patients and χ^2^ tests to compare the prevalence of categorical variables within each of the two groups. Survival analyses of deregulated metabolic genes in male and female patients were conducted using the survival package (version 2.41–3; https://CRAN.R-project.org/package=survival) in R. Each gene was divided into two groups (high and low) according to median expression values. Kaplan-Meier survival curves were used to express differences between high and low expression levels of each metabolic gene among groups in relation to the prognosis of the patient. The Cox proportional hazards model was used to explore the association between metabolic genes and the prognosis of patients and P ≤ 0.05 was considered statistically significant. Metabolic cancer susceptibility genes were defined as being either: (1) upregulated in tumor samples and high expression was associated with reduced prognosis or (2) downregulated in tumor samples and low expression was associated with reduced prognosis. Robust likelihood-base survival analysis in "rbsurv" package in R was used to construct the combination model of metabolic cancer susceptibility genes on patients prognosis in male and female. The method was chose as "efron" and the maximum number of genes considered was set as 10.

### Protein-protein interaction networks and ROC curves of risk metabolic genes

We used STRING web server (https://string-db.org/cgi/input.pl) [17] to construct protein-protein interaction (PPI) networks of metabolic cancer susceptibility genes and other related genes in male and female patients. The parameters were set as follows: (1) the network edges were set as the molecular action (line shape indicates the predicted mode of action); (2) all types of active interaction sources were chosen, including text mining, experiments, databases, co-expression, neighborhood, gene fusion, and co-occurrence; (3) the minimum required interaction score was set at the medium confidence of 0.4; (4) the maximum number of interactors in the first shell was the total number of query proteins only and the maximum number of interactors in second shell was set to no more than 20. Then the biological functions of the selected metabolic cancer susceptibility genes were automatically exported from the web server. We used the pROC package (version 1.12.1) in R [18] to display and analyze the receiver operating characteristic (ROC) curves of the metabolic cancer susceptibility genes in male and female patients. The expected power of the test (probability of type II error) was calculated for each gene.

### Patients and tissue samples

The study was approved by the Medical Ethics Committee of the First Affiliated Hospital of Kunming Medical University. All patients voluntarily joined this study and each provided an informed consent form. Eight patients(four males and four females were age matched, with a maximum age of 71 years, a minimum age of 55 years, and a median age of 65 years) diagnosed with non-small cell lung adenocarcinoma cancers (NSCLCs) at the first affiliated hospital of Kunming Medical University were enlisted(tumor histology was estimated by at least 2 pathologists). The 8 paired samples collected were used to verify analysis. The patients enrolled met the following criteria: (1) The patient was diagnosed as lung adenocarcinoma by pathology; (2) the patients had not received radiotherapy or chemotherapy; (3) tumor and adjacent normal lung tissues (> 5 cm away from carcinoma tissues) were obtained. The tissue samples were collected at the time of surgery and then quickly frozen in liquid nitrogen until further use. The tumor samples contained a tumor cellularity greater than 80% and the matched control samples had no cancer cells.

### RNA isolation and quantitative real-time PCR (qRT-PCR)

RNA was extracted from 8 paired lung adenocarcinoma samples (4 pairs of males and 4 pairs of females) using the AllPrep DNA/RNA Mini Kit (Qiagen, Germany), and cDNA was generated using PrimeScript Reverse Transcriptase (Fermentas K1622, USA). qPCR was performed using the SYBR Green master mix (KAPA KK4601, USA) and the Applied Biosystems 7300 RealTime PCR System (Applied Biosystems, USA) was used for the analysis. The real-time PCR utilized the following primers: 5′- CAC CTC AAC ACA ATT CAG -3′ (forward) and 5′- TTC TCT TCG CTT ATT ATA TTC C-3′ (reverse) for TAOK1, 5′- AAT AGC ACA AGT TAT GAA G-3′ (forward) and 5′- TAT ACA TCC AAT GAT TCC T-3′ (reverse) for ASAH1, 5′-AAA GGG TCA TCA TCT CTG -3′ (forward) and 5′-GCT GTT GTC ATA CTT CTC -3′ (reverse) for GAPDH. PCR was performed under the following conditions: 50°C 2 min, 95°C for 10 minutes, 95°C for 15 seconds and 60°C for 30 seconds for 45 cycles. Relative expression of TAOK1 and ASAH1 mRNA level was calculated by the comparative CT method.

### Western blotting

The cell lysate was prepared from 8 paired lung adenocarcinoma tumor and adjacent tissues using RIPA lysis buffer (Solarbio, China) containing protease inhibitor (Millipore, USA). The protein concentration was measured using a BCA protein assay (Solarbio, China). Approximately 60 μg protein samples were isolated in 10% acrylamide gel and transferred to PVDF membranes. The membranes were blocked for 1 hours at room temperature with 5% BSA. Membranes then were incubated with rabbit anti-TAOK1 and rabbit anti-ASAH1 at 1:2000 dilution (Abcam, USA) for overnight at 4°C. Mouse anti-β-actin (Abcam, USA) was used to normalize the amount of sample loaded. Next, the nonspecific binding of primary antibody was washed out and secondary antibody (goat-anti-rabbit HRP and goat-anti-mouse HRP) was added and incubated for 1 hour at room temperature. Later the signals are visualized with a chemiluminescent substrate reagent kit (Thermo Fisher Scientific, USA). The band intensity was measured by Image J software(v1.8.0).

### Constructs and cell culture

The sequence of TAOK2 and ASAH1 was obtained from Genbank. For TAOK2,due to its low expression, Pez-lv105 expression vector was used to construct the overexpressed plasmid(ex-TAOK2),blank vector (exCtrl); For ASAH1, due to its high expression, according to Oligoengine online software analysis results, the two optimal siRNA target sequences without homology to genes of other mammals was screened out and pSi-LVRU6P expression vector was used to construct the knockdown lasmid(siASAH1#1 and siASAH1#2), blank vector (siCtrl). These plasmids were provided by the Guangzhou FulenGen (Guangzhou,China).Lentiviruses were generated according to the manufacturer’s protocol; cells were infected by viruses twice for 48 and 72 h with viral supernatants containing 4μg/ml polybrene.HEK-293T cells were obtained from ATCC and cultured following standard protocol. A549 and XWLC-05 lung adenocarcinoma cancer cell lines were gifts from the Institute of Clinical Cancer, Kunming Medicine University, A549 and XWLC-05 cells were all cultured in RPMI-1640 medium (Hyclone, USA) supplemented with 10% fetal bovine serum (FBS) and 1% penicillin/streptomycin. All cells were cultured at 37°C in a 5% CO2 humidified environment.

### Cell proliferation

Stable transfection A549 and XWLC-05 cells were seeded in 96-well culture plates, About 2,000 cells were inoculated per well. CCK-8 (Beyotime, Beijing, China) was used to perform the cell proliferation analysis according to the manufacturer’s instructions. Repeat each experiment three times.

### Cell migration and invasion assay

To conduct a wound-healing migration assay, the stable transfection cells were seeded onto 35 mm dishes coated with fibronectin. Once the cells had reached 100% confluence, a scratch was created on the confluent monolayer using a sterile 200ul pipette tip. The cell debris was then removed by replacing the medium with fresh serum-free medium. During the subsequent 24 hours culture of the cells, the width of the wound was measured at 0 hours and 24 hours time points. Three to four different locations were visualized and photographed under a phase-contrast inverted microscope.

Stable transfection A549 and XWLC-05 cells were inoculated in the upper transwell chambers (Corning, USA) with serum free media. The density of the cells was 3×10^4^/ml,and each well had three parallels (200μl/well). 500μl culture media containing 10% FBS was placed in lower chambers at 37°C (5% CO2). After the culture for 48 h, the cells in upper chambers were removed with swab, the cells on the bottom side of the upper chambers were exposed to 400μl. crystal violet (0.1%) dye for 10 min. The cells of 5 random horizons were observed and photographed under a microscope.

### Statistical analysis

All quantitative data were presented as mean ± SD. Paired-sample t-test was applied to compare mRNA expression level between tumor tissue and matched adjacent non-tumor tissue. Wilcoxon rank sum test was used for comparisons of protein level in two independent groups. Statistical analysis was performed by using the Statistical Package for Social Sciences (SPSS) software (version 22.0). Statistical significance was accepted at P < 0.05.

## Results

### Overall differences in metabolic gene expression and clinical features grouped by gender

**Fig 1:**
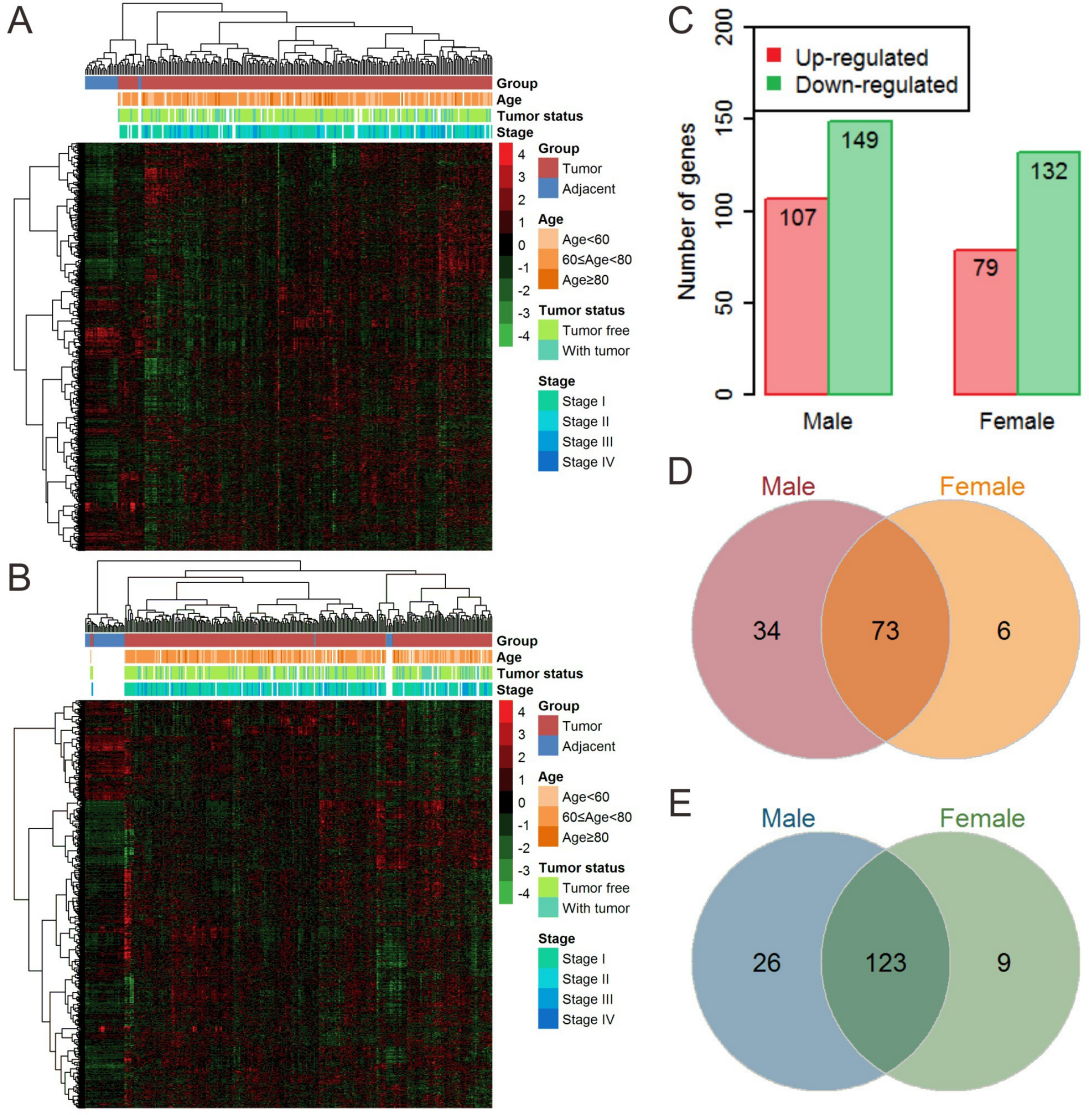
Overall metabolic gene expression profiles. (A) Heatmap of metabolic genes in males. (B) Heatmap of metabolic genes in females. (C) Number of upregulated and downregulated genes in males and females. (D) Venn diagram of upregulated genes in males and females. (E) Venn diagram of downregulated genes in males and females. All expression values were converted to z-scores.

No differences in the distribution of age, race, tumor status, or stage of caner between male and female patients were observed (Table 1). However, more male patients were smokers compared to female patients (P < 0.001). Through hierarchical clustering, we found large differences in metabolic gene expression profiles between tumor samples and adjacent normal samples for both male and female patients (Fig 1A and 1B). Moreover, a higher proportion of metabolic genes were upregulated or downregulated in male patients compared to females (Fig 1C). Furthermore, Venn diagrams were used to depict 73 commonly upregulated and 123 downregulated metabolic genes between male and female patients (Fig 1D and 1E). Furthermore, we observed that most of the absolute value of the log2(FC) were higher in male (tumor vs. adjacent) than female (tumor vs. adjacent), suggested that the deregulation level of metabolic genes in male were greater than in females (S1 and S2 Tables).

**Table 1:** Descriptive statistics of the clinical characteristics of LUAD patients stratified by gender.

Variables	Male	Female	P
Age	65.5 ± 9.8	65.1 ± 10.3	0.700
Race			
White	172 (86.4)	215 (86.7)	0.739
Asian	4 (2.0)	3 (1.2)	
Black or African American	23 (11.6)	29 (11.7)	
American Indian or Alaska Native	0 (0.0)	1 (0.4)	
Smoking history			
Non-smoker	20 (8.7)	54 (20.1)	< 0.001
Former smoker	142 (61.7)	163 (60.6)	
Current smoker	68 (29.6)	52 (19.3)	
Tumor status			
Tumor free	158 (75.6)	182 (73.1)	0.615
With tumor	51 (24.4)	67 (26.9)	
Stage			
Stage I	113 (48.7)	161 (59.0)	0.059
Stage II	67 (28.9)	54 (19.8)	
Stage III	38 (16.4)	46 (16.8)	
Stage IV	14 (6.0)	12 (4.4)	

### Metabolic subsystem changes in male versus female tumors

**Fig 2:**
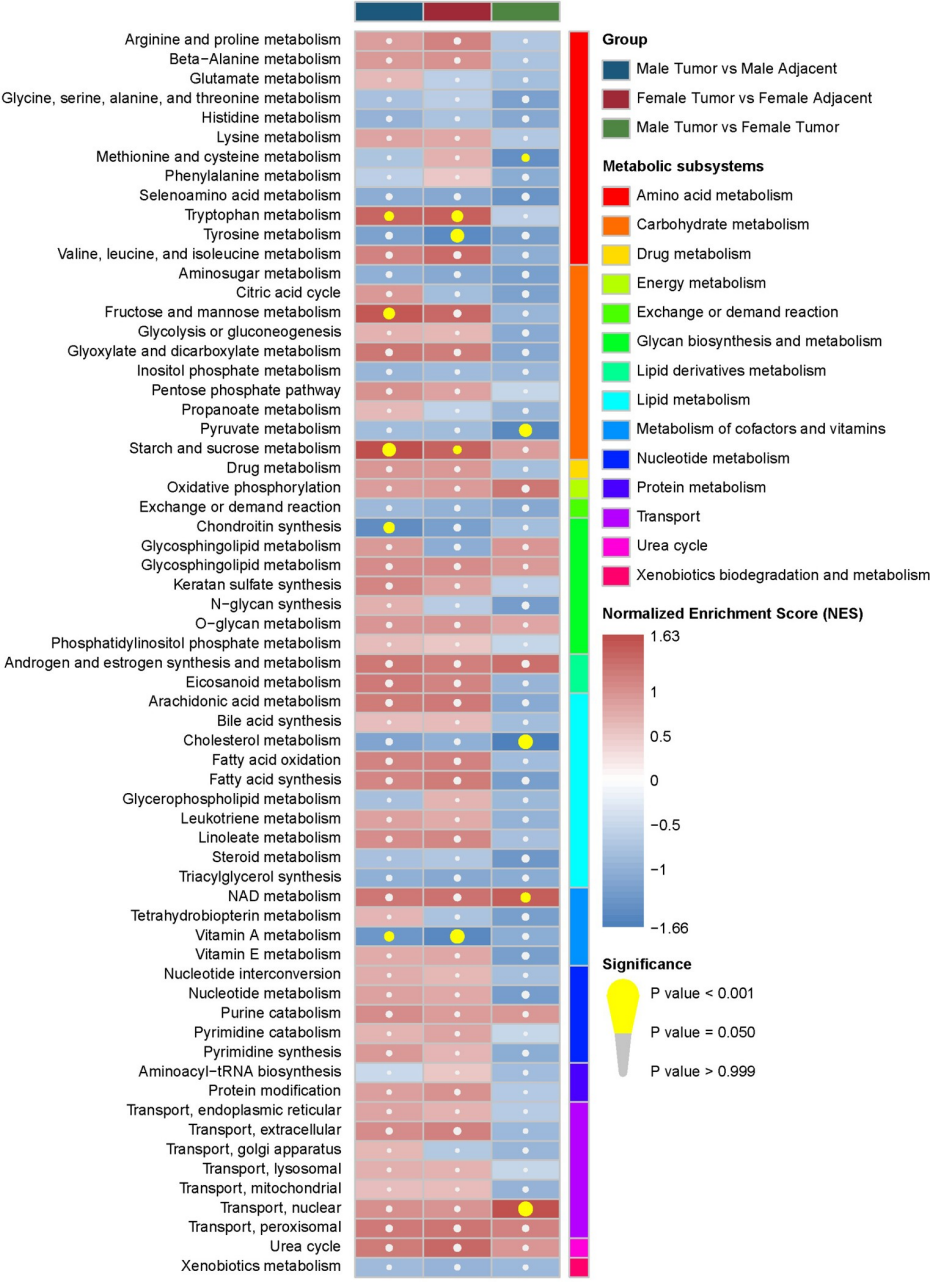
Gene set enrichment analysis results of enriched metabolic subsystems. Metabolic subsystems with more than 15 genes are shown (represented by different colors) based on three comparisons (male tumor vs male adjacent, female tumor vs female adjacent, male tumor vs female tumor). The red box represents the metabolic subsystem that is upregulated, and the blue box represents the metabolic subsystem that is downregulated. The yellow circle indicates that the metabolic subsystem is significantly enriched.

The enriched metabolic subsystems within male tumor vs. male adjacent, female tumor vs. female adjacent, and male tumor vs. female tumor are presented in Fig 2. The results show that tryptophan metabolism as well as starch and sucrose metabolism were upregulated in both male and female tissues, and vitamin A metabolism was downregulated. Furthermore, tyrosine metabolism was downregulated in female but not in male tumor tissues, whereas chondroitin synthesis was downregulated in male but not in female tissues. A comparison of male tumor vs. female tumor samples suggests three metabolic subsystems (methionine and cysteine metabolism, pyruvate metabolism, cholesterol metabolism) are downregulated and two metabolic subsystems (NAD metabolism and transport, nuclear) are upregulated. However, these metabolic subsystems were no statistical significance after FDR correction (S1 Fig).

### Prognosis-correlated metabolic genes and risk metabolic genes screen

**Fig 3:**
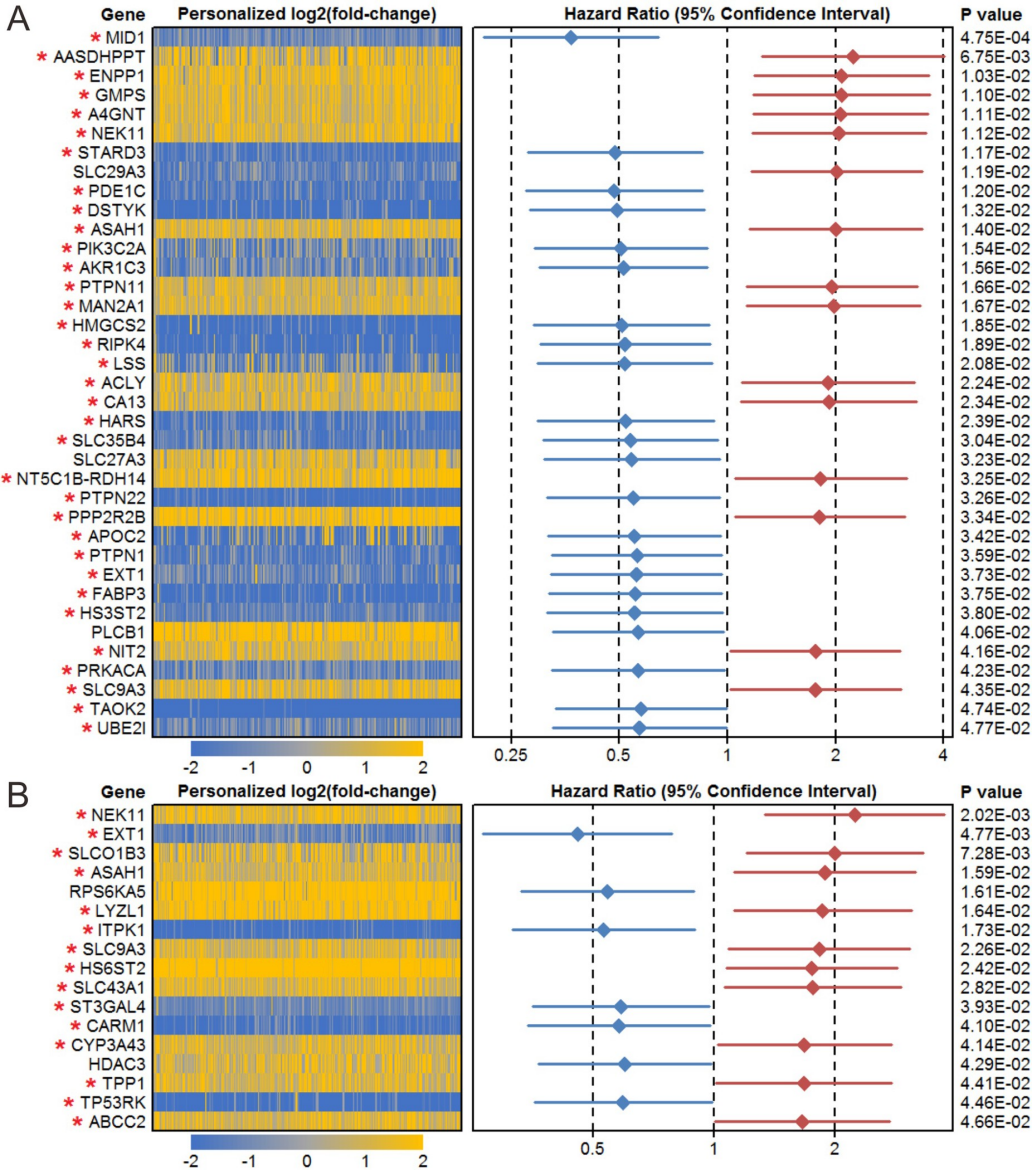
Prognosis-correlated metabolic genes in males (A) and females (B). Personalized fold changes (log2(FC)) were calculated as the gene expression values for each patient/mean expression of the gene in controls. All upregulated (yellow) and downregulated (blue) genes were deregulated. Metabolic cancer susceptibility genes are labeled with a red asterisk.

We performed survival analyses using deregulated metabolic genes in male and female patients. The results show that 37 deregulated metabolic genes affect the prognosis of male patients and 17 affect the prognosis of female patients (Fig 3 and S3 and S4 Tables). We further analyzed the effect of menopause on differential expression. Based on the previous report, female > 50 years were defined as menopause group [19]. The results showed a consistent difference between premenopausal and menopause group (S5 Table). High expression of ASAH1, NEK11, and SLC9A3 and low expression of EXT1 were also linked to reduced prognosis in both male and female patients (S2 Fig). Other genes were also shown to influence the prognosis of male and female patients differently (S3 Fig). Based on the definition of metabolic cancer susceptibility genes, we screened 34 genes in male patients and 15 genes in female patients. The multi-gene combination model suggested that the combination of NEK11, HS3ST2, ACLY, HARS and SLC35B4 is the best model for male prognosis prediction, and the combination of TP53RK, TPP1, ST3GAL4, LYZL1, ITPK1, ASAH1 and CYP3A43 is the best model for female prognosis prediction (S6 Table).

### Biological functions and ROC curves of risk metabolic genes

**Fig 4:**
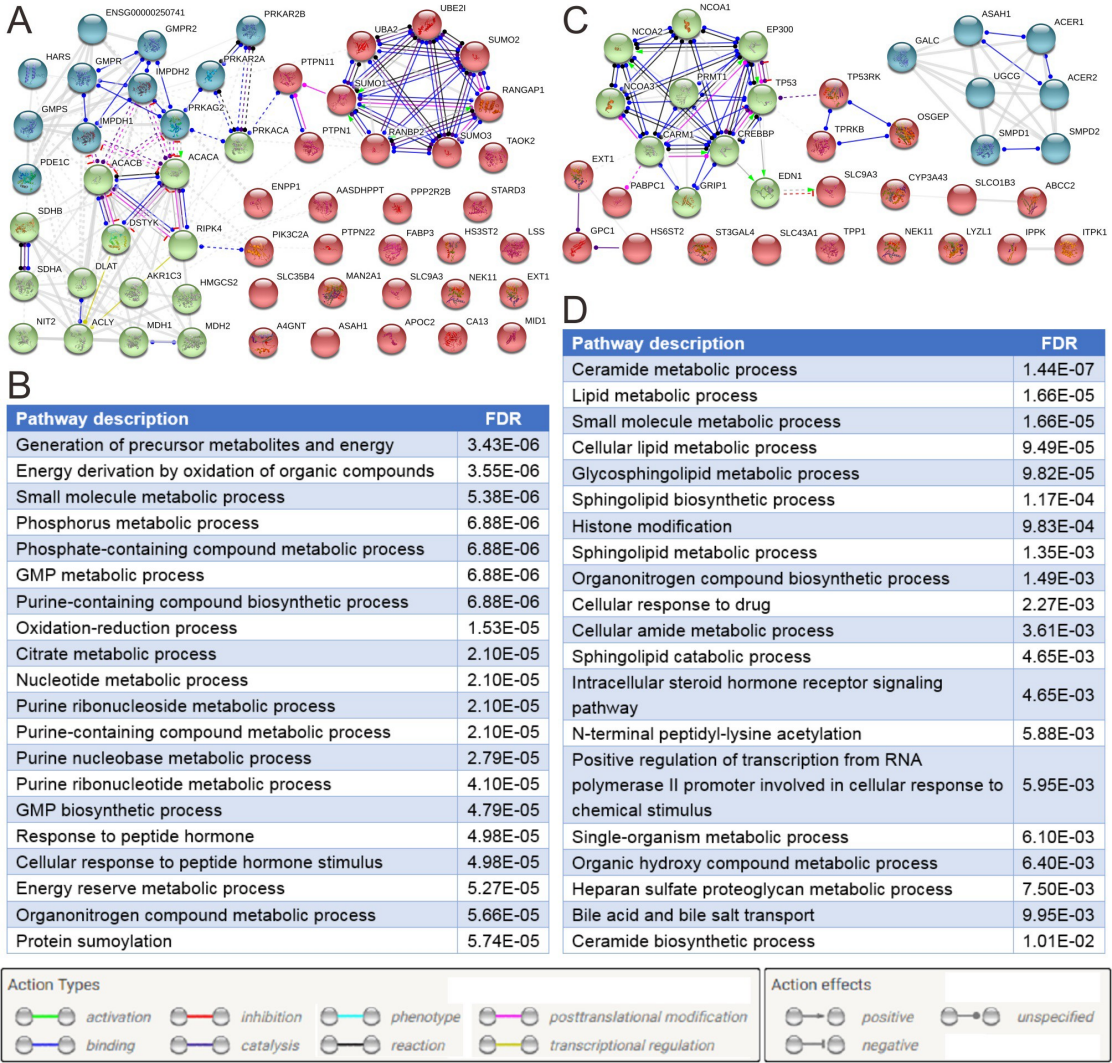
Protein-protein interaction (PPI) networks of risk metabolic genes and related genes. (A) PPI network of 34 risk metabolic genes and related genes in male patients. (B) Top 20 enriched biological functions of risk metabolic genes and related genes in male patients. (C) PPI network of 15 metabolic cancer susceptibility genes and related genes in female patients. (D) Top 20 enriched biological functions of metabolic cancer susceptibility genes and related genes in female patients. The k-means clustering method was used and up to 3 specified groups were clustered.

**Fig 5:**
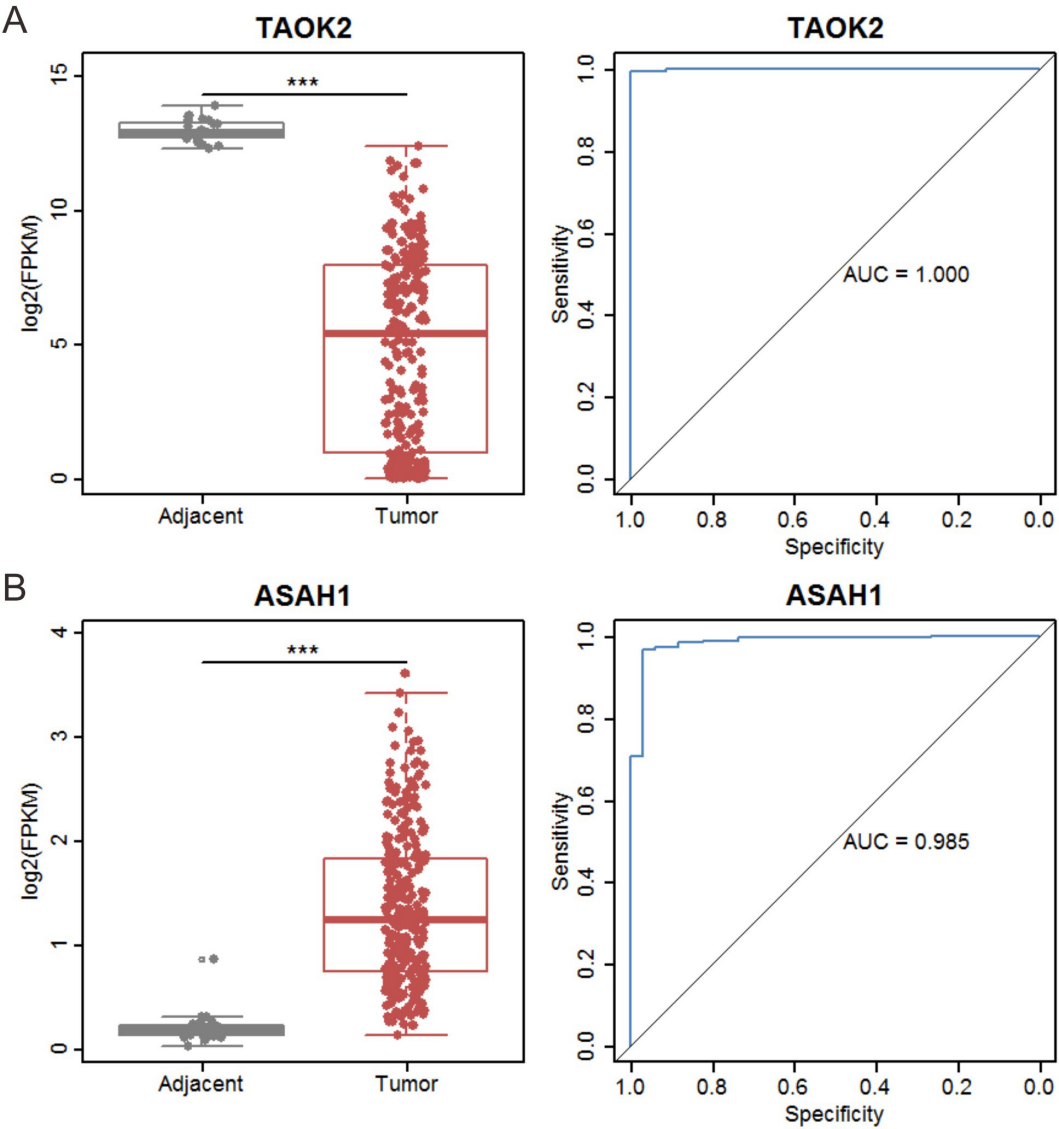
Expression and ROC curves of TAOK2 and ASAH1. (A) Differences in expression (left panel) and ROC curves and AUC (right panel) of TAOK2 in male patients. (B) Differences in expression (left panel) and ROC curves and AUC (right panel) of ASAH1 in female patients.

Protein-protein interaction networks of metabolic cancer susceptibility genes and related genes in males and females are presented in Fig 4. The results suggest these genes are mainly involved in nucleotide metabolism and energy metabolism in male patients, whereas they are mainly involved in the metabolism of lipids and lipid derivatives in female patients. We performed ROC curve analyses for all metabolic cancer susceptibility genes in male and female tumor tissues. The area under the curve (AUC) values of these genes in male and female tissues are listed in S7 and S8 Tables. Among these genes, TAOK2 showed the highest diagnostic accuracy in male and ASAH1 showed the highest diagnostic accuracy in female both in whole cohort and in early stage (stage I and II) patients. Interestingly, TAOK2 was downregulated in males whereas ASAH1 was upregulated in females (Fig 5).

### Verification of metabolic cancer susceptibility genes using microarray datasets

We performed further analyses to verify the expression of metabolic cancer susceptibility genes and their effects on the prognosis of patients. Considering the large heterogeneity between microarray data and sequencing data, we mainly focused on the differential expression trends (expression of metabolic cancer susceptibility genes in cases vs. controls) and prognosis trends (effect of risk metabolic genes on the overall survival of patients) in two independent datasets. There were 33 and 14 overlapping metabolic cancer susceptibility genes between GSE72094 dataset and TCGA data for male and female groups, respectively. Moreover, there were 31 and 12 overlapping risk metabolic genes between GSE68465 dataset and TCGA data for the male and female groups, respectively. We found the same prognosis trends (high expression of the gene increase/decrease patient survival both in TCGA data and validation set) in male and female patients for 61% and 43% of metabolic cancer susceptibility genes based on the GSE72094 dataset (S9 Table). Furthermore, 48% and 67% of the metabolic cancer susceptibility genes in the GSE68465 dataset exhibited similar expression trends in male vs. female groups, and 55% and 42% of genes showed similar trends in the prognosis of male and female patients (S10 Table). There were 4 genes in male (LSS, PPP2R2B, PRKACA and TAOK2) and 2 genes in female (CARM1 and SLCO1B3) were both expression and prognosis validated in the two microarray datasets. Furthermore, we also performed GSEA of enriched metabolic subsystems in GSE68465 dataset. However, the results showed that there was a opposite trend in the GSE68465 dataset compared with the TCGA dataset (S4 Fig). The validation of multi-gene combination model suggested that the combination of ASAH1 and other genes can accurately predict patients prognosis both in male and female patients (S11 Table).

### Expression of TAOK2 and ASAH1 in human lung tissues

**Fig 6:**
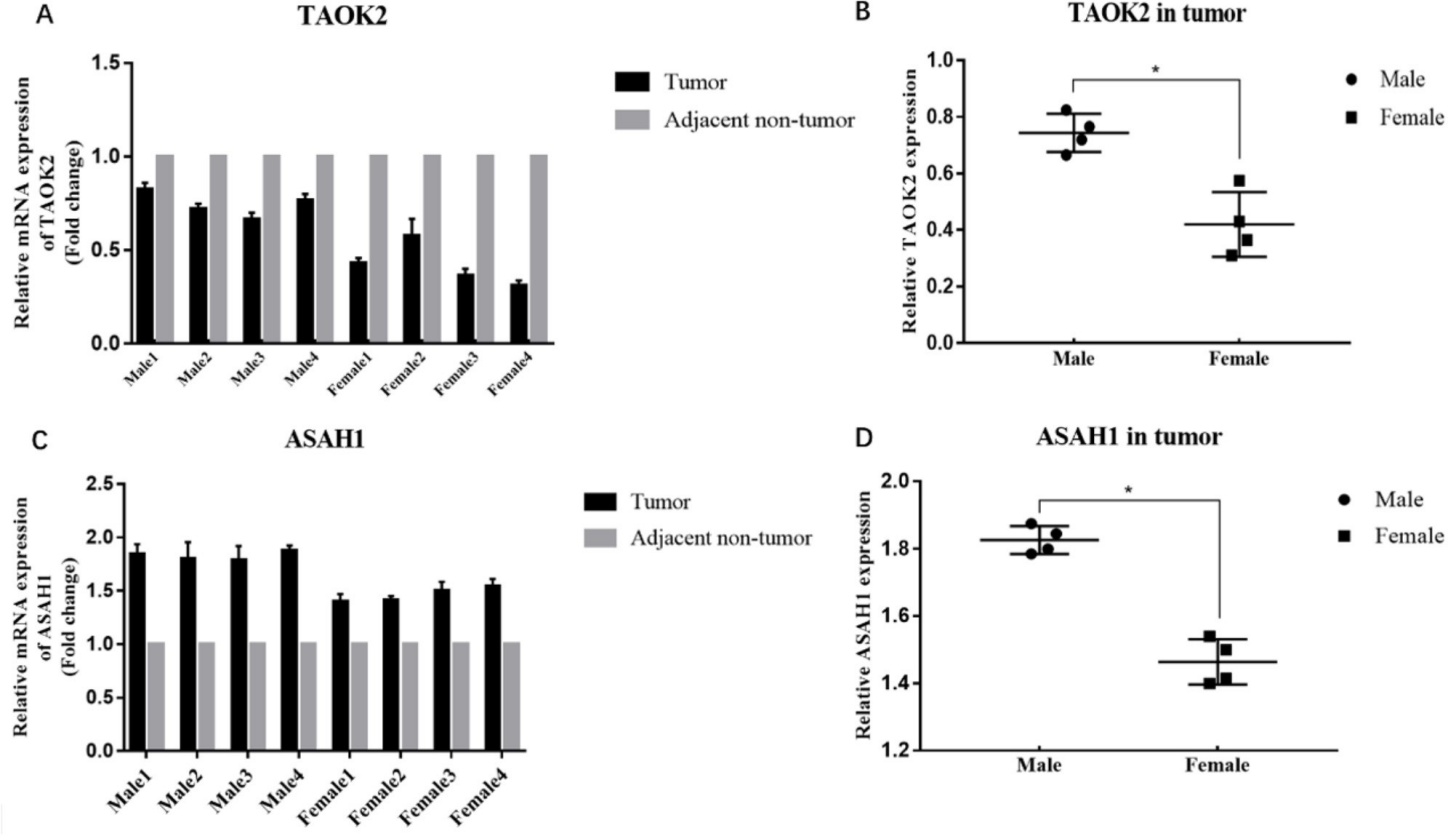
Identification of down-regulated TAOK2 and up-regulated ASAH1 in lung adenocarcinoma. (A) TAOK2 mRNA expression decreased in tumor tissue. (B) The expression of TAOK2 in male tumor tissue and female tumor tissue. (C) ASAH1 mRNA expression decreased in tumor tissue. (D) The expression of ASAH1 in male tumor tissue and female tumor tissue. (* P < 0.05).

**Fig 7:**
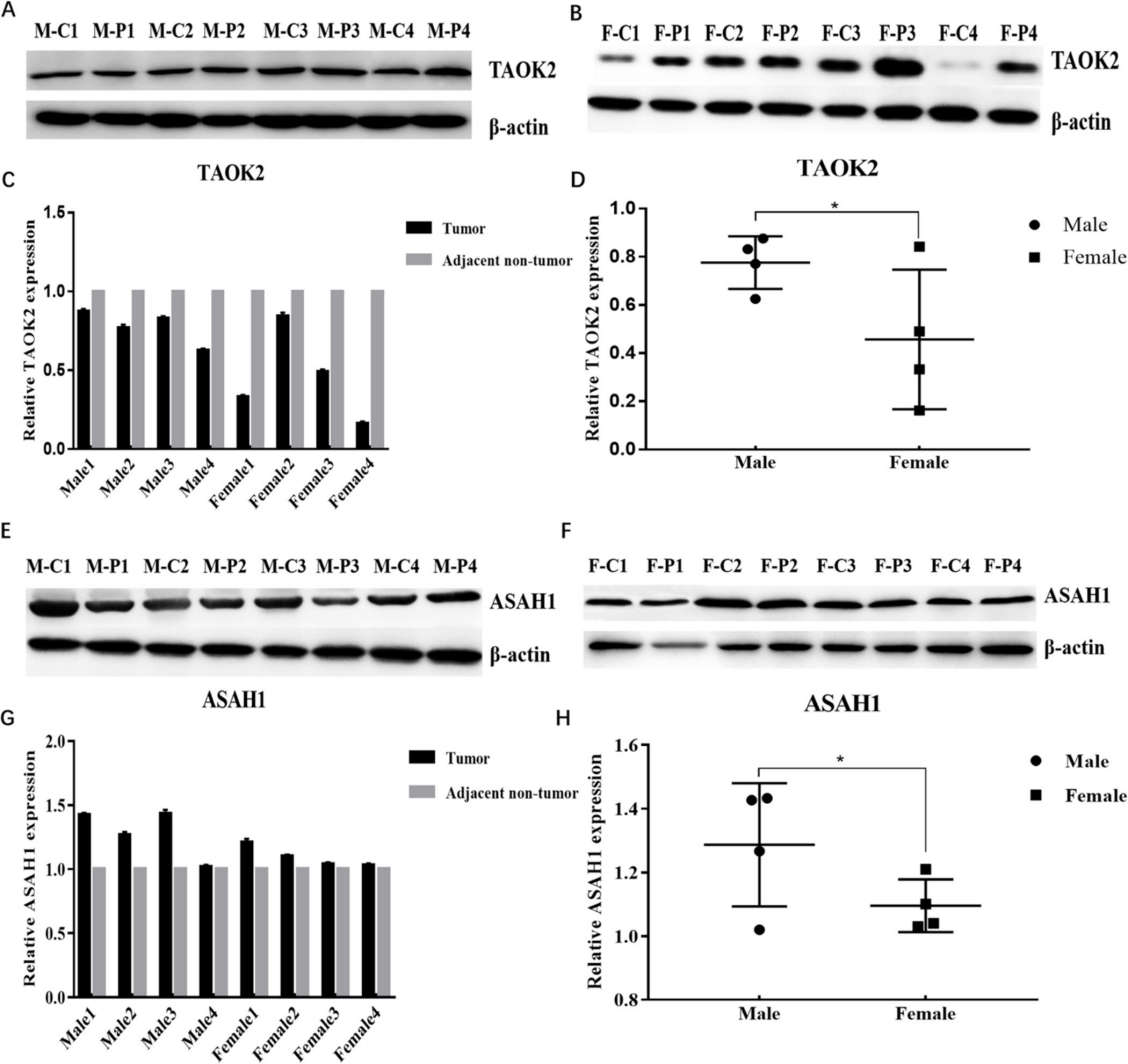
Identification of down-regulated the protein expression of TAOK2 and up-regulated ASAH1 in lung adenocarcinoma by Western blotting (n = 8). (A) The TAOK2 protein expression decreased in male tumor tissue. (B) The TAOK2 protein expression decreased in female tumor tissue. (C) TAOK2 protein expression decreased in tumor tissue compare to adjacent tissue in male and female. (D) TAOK2 protein expression increased in male tumor tissue compare to female tumor tissue. (E) The ASAH1 protein expression increased in male tumor tissue. (F) The ASAH1 protein expression increased in female tumor tissue. (G) ASAH1 protein expression increased in tumor tissue compare to adjacent tissue in male and female. (H) ASAH1 protein expression increased in male tumor tissue compare to female tumor tissue. M-C—Male lung adenocarcinoma tissue, M-P—Male matched adjacent non-tumor tissue, F-C—Female lung adenocarcinoma tissue, F-P—Female matched adjacent non-tumor tissue. (* P < 0.05).

To identify TAOK2 *and* ASAH1 expression difference in male and female tumor tissue in lung adenocarcinoma, we collected 8 pairs of tissues (tumor tissue and matched adjacent non-tumor tissue, 4 males and 4 females) by quantitative reverse transcription polymerase chain reaction (qRT-PCR). Results revealed that TAOK2 mRNA was down-regulated and ASAH1 mRNA was up-regulated in lung adenocarcinoma in male tumor tissue and female tumor tissue. However, TAOK2 and ASAH1 mRNA expression were higher in men than in women (P < 0.05, Fig 6). Next, we further examined the protein expression of TAOK2 and ASAH1 in 8 pairs of tissues by Western blotting. As expected, in general, TAOK2 protein was suppressed and ASAH1 protein was increased in lung adenocarcinoma in male tumor tissue and female tumor tissue. However, TAOK2 and ASAH1 protein expression were higher in men than in women, the difference remained statistically significant (P < 0.05, Fig 7).

### Functional analysis of ASAH1 and TAOK2 in lung adenocarcinoma cells

**Fig 8:**
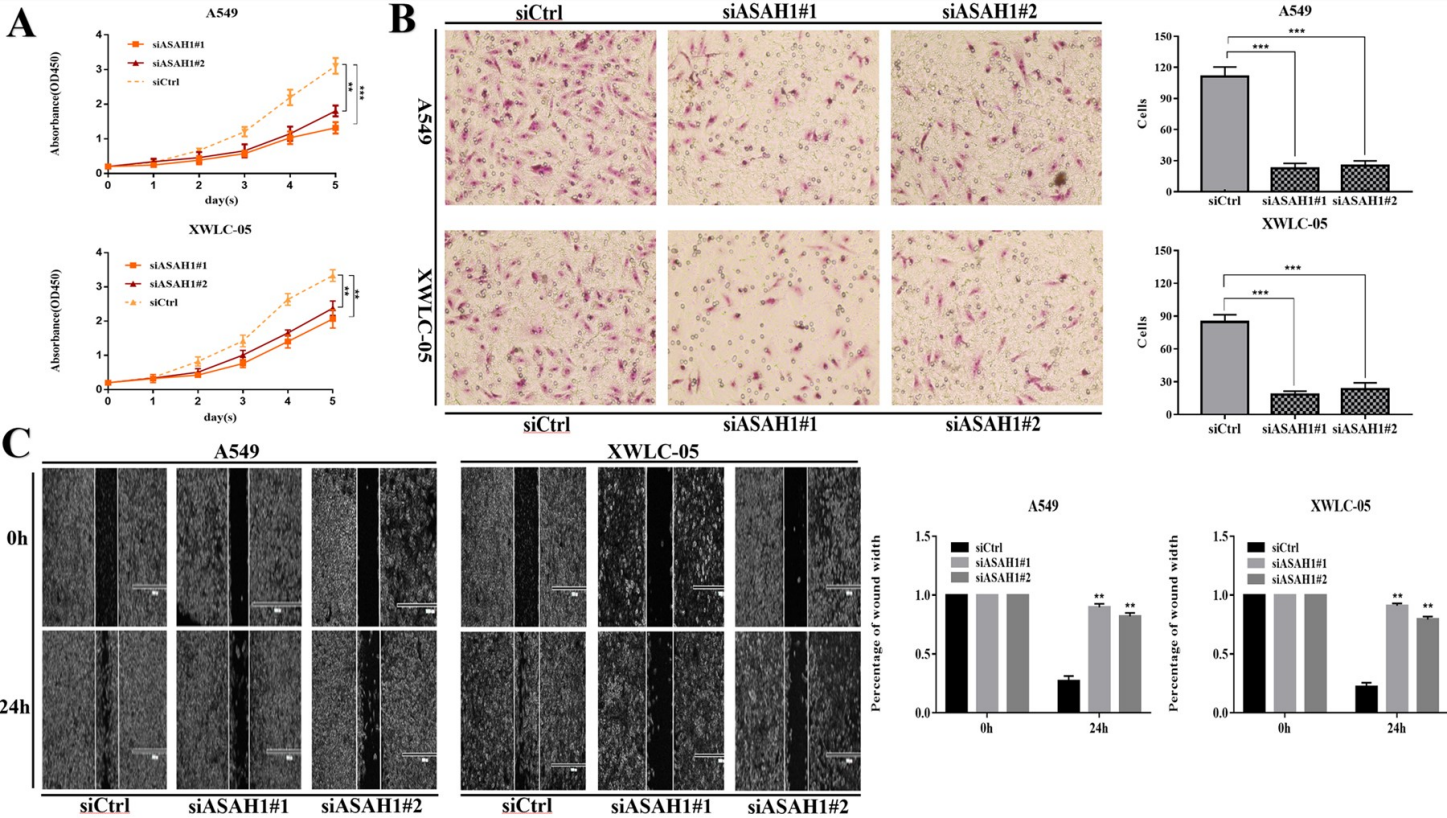
Effect of downregulation of ASAH1 on the proliferation, migration, and invasion of lung adenocarcinoma cancer cells in vitro. (A) The CCK-8 assay results showing the proliferation ability of A549 and XWLC-05 cells. Cells were transfected with knockdown plasmid (siASAH1#1 and siASAH1#2), blank vector (siCtrl). (B) The invasion assay results showing the invasion ability of A549 and XWLC-05 cells. The results were from three independent experiments. The cell number in each group was normalized to the control. Cells were transfected with knockdown plasmid (siASAH1#1 and siASAH1#2), blank vector (siCtrl). (C) The wound-healing assay results showing the migration ability of A549 and XWLC-05 cells. Cells were transfected with knockdown plasmid (siASAH1#1 and siASAH1#2), blank vector (siCtrl) and photos were taken in 40x field of vision. Images were taken in 40x field of vision. (*P < 0.05,**P < 0.01,***P < 0.001, Student’s t-test).

The functional significance of ASAH1 and TAOK2 in lung cancer cells was evaluated. Briefly, A549 and XWLC-05 cells with high ASAH1 expression or low TAOK2 expression were transfected with knockdown plasmid (siASAH1#1 and siASAH1#2) and overexpression plasmid(ex-TAOK2) or blank vector (siCtrl or exCtrl), respectively. CCK-8 assay and invasion assay were performed to investigate the effect of ASAH1 and TAOK2 on cell proliferation and cell invasion, respectively. Wound-healing assay was used to examine cell migration ability. The results showed that the proliferation ability of A549 and XWLC-05 cells transfected with knockdown plasmid (siASAH1#1 and siASAH1#2) was lower than that of the control group (Fig 8A), and the invasion ability of A549 and XWLC-05 cells transfected with knockdown plasmid was lower than that of the control group (Fig 8B). Furthermore, the downregulation of ASAH1 in A549 and XWLC-05 significantly inhibited cell migration compared to the control group (Fig 8C). Collectively, these results suggest that ASAH1 could be an oncogene, and thus inhibiting ASAH1 can suppress the proliferation, migration, and invasion of lung cancer cells. However, overexpression of TAOK2 had no significant effect on cell proliferation, migration and invasion ability both in A549 and XWLC-05 cells (S5 Fig).

## Discussion

A large-scale cancer transcriptome study recently revealed multiple abnormalities in the metabolic function of various cancer cells using a personalized genome-scale metabolic modeling approach [20]. The same study found that genes associated with metabolic damage may also affect the prognosis of patients. Our previous research suggests key metabolic genes play crucial roles in kidney cancer [21] and liver cancer [22]. In the present study, we revealed gender-specific metabolic changes in LUAD patients. By integrating the LUAD transcriptome, clinical data, and metabolic information, we were able to identify 34 metabolic cancer susceptibility genes in male patients and 15 in female patients. The expression profiles of these risk metabolic genes in patients predicted poor overall survival.

Dysregulation of cellular metabolism promotes tumor aggressiveness by sustaining the activity of key growth, invasion, and survival pathways. Previous studies have identified several key metabolic pathways (glycolysis, glutamine metabolism, oxidative phosphorylation, et al.) and the metabolic genes involved in these pathways as potential therapeutic targets [23–25]. Cancer cells are reprogrammed to consume large amounts of glucose to support anabolic biosynthetic pathways. A recent study reported that silencing of phosphoenolpyruvate carboxykinase mitochondrial (PEPCK-M) isoforms can suppress cancer growth in a lung cancer cell xenograft model [26].

A high aerobic glycolysis rate is another characteristic of tumor metabolism. Evidence from multiple studies suggests that lactate dehydrogenase activity in serum may reflect the glycolytic activity of tumor cells and thus acidity within the tumor microenvironment [27]. Furthermore, systemic oxidative stress is associated with the pathogenesis of lung cancer and many other site-specific cancers. Clinical research suggests circulating glucose or non-enzymatic glycation are correlated with oxidative stress, whereas metabolites such as β-hydroxybutyrate and non-esterified fatty acids are linked to total antioxidant status in lung cancer patients [28]. In this study, we found that multiple pathways of amino acid and carbohydrate metabolism were deregulated both in male and female patients. Some major metabolic pathways showed large heterogeneity in male vs. female patients. Therefore, we speculate that the regulation of major metabolic systems such as amino acid metabolism, glucose metabolism, and lipid metabolism may be gender-specific in LUAD patients.

The present study identified several metabolic cancer susceptibility genes involved in different biological functions in male and female lung cancer patients. Through ROC curve analysis, we found that TAOK2 is crucial for male patients’ prognosis and ASAH1 play an important role in female patients. TAOK2 is a serine/threonine protein kinase that has been implicated in neurodevelopmental disorders. Studies show that TAOK2 heterozygous and KO mice have dosage-dependent abnormalities in brain size and neural connectivity in multiple regions [29]. TAOK1 and TAOK2 are catalytically activated during mitosis and can contribute to mitotic cell rounding and spindle positioning. A recent study showed that TAOK inhibition prolongs the duration of mitosis in breast cancer cells, increases mitotic cell death, and reduces the percentage of cells exiting mitosis [30]. Furthermore, genetic analyses identified a rare TAOK2 homozygous missense variant that causes a novel form of primary immunodeficiency [31].

High-throughput screening has identified TAOK2 as a potential cancer therapeutic target and three specific small molecule compounds were found to inhibit TAOK2 [32]. ASAH1 encodes a member of the acid ceramidase family of proteins and catalyzes the hydrolysis of ceramide into sphingosine. In turn, a substrate of sphingosine kinases catalyzes the conversion of sphingosine into mitogenic sphingosine-1-phosphate [33]. The ASAH1 enzyme is overexpressed in multiple human cancers and may promote cancer progression [33–36]. Stable knockdown of ASAH1 in a prostate cancer cell line (PC-3/Mc) caused accumulation of ceramides, an increased requirement for growth factors, and inhibition of tumor cell proliferation and migration [33]. In breast cancer, over-expressed ASAH1 was found in estrogen receptor (ER)-positive patients compared to ER-negative patients. However, high expression of ASAH1 was associated with improved prognosis in invasive breast cancer [34]. High expression of ASAH1 was also found in human colon cancer cells and colorectal adenocarcinoma tissues and was shown to be negatively correlated with p53 functional activity in tumor cells [35]. Furthermore, low ASAH1 expression was associated with invasive behavior of melanoma cells and therefore, may present a new therapeutic target [37]. Further, through function experiments in lung adenocarcinoma cell lines in vitro, we found that ASAH1 could be an oncogene, and thus inhibiting ASAH1 can suppress the proliferation, migration, and invasion of lung cancer cells.

This study has some limitations. First, the causal relationship between metabolic cancer susceptibility genes and lung cancer progression remains unclear. The current results can only prove a correlation between these genes and lung cancer. Second, lung cancer metabolism may be affected by a variety of environmental factors and lifestyle habits; however, owing to a lack of data, we were unable to conduct further analysis in this area. Third, the validation data set can only explain about half of the findings. Further experiments are still needed to fully verify our analysis.

## Conclusions

In conclusion, this study has revealed the overall metabolic differences between male and female tissues in LUAD. We identified 34 metabolic cancer susceptibility genes in males and 15 in females, which all have high AUC values, suggesting that these genes, alone or in combination, may be potential diagnostic markers for LUAD. This study provides valuable information for the molecular diagnostics in LUAD and future research studies.

## Supporting information

S1 Fig Gene set enrichment analysis results of enriched metabolic subsystems. All P values were corrected using FDR. The red box represents the metabolic subsystem that is upregulated, and the blue box represents the metabolic subsystem that is downregulated. The yellow circle indicates that the metabolic subsystem is significantly enriched.
(DOCX)

S1 Table Commonly up-regulated metabolic genes in male and female. (DOCX)

S2 Fig Commonly risk metabolic genes in male and female patients. (DOCX)

S2 Table Commonly down-regulated metabolic genes in male and female. (DOCX)

S3 Fig Example of different effect of risk metabolic genes on prognosis in male and female patients. (DOCX)

S3 Table Effect of 37 deregulated metabolic genes on male prognosis. (DOCX)

S4 Fig Gene set enrichment analysis results of enriched metabolic subsystems in validation dataset (GSE68465). The red box represents the metabolic subsystem that is upregulated, and the blue box represents the metabolic subsystem that is downregulated. The yellow circle indicates that the metabolic subsystem is significantly enriched.
(DOCX)

S4 Table Effect of 17 deregulated metabolic genes on female prognosis. (DOCX)

S5 Fig Effect of overexpression of TAOK2 on the proliferation, migration, and invasion of lung adenocarcinoma cancer cells in vitro. (A) The CCK-8 assay results showing the proliferation ability of A549 and XWLC-05 cells. Cells were transfected with overexpression ex-TAOK2 plasmid or blank vector (exCtrl).(B) The invasion assay results showing the invasion ability of A549 and XWLC-05 cells. The results were from three independent experiments. The cell number in each group was normalized to the control. Cells were transfected with overexpression ex-TAOK2 plasmid or blank vector (exCtrl).(C) The wound-healing assay results showing the migration ability of A549 and XWLC-05 cells. Cells were transfected with overexpression ex-TAOK2 or blank vector (exCtrl) and photos were taken in 40x field of vision. Images were taken in 40x field of vision.(*P < 0.05,**P < 0.01,***P < 0.001, Student’s t-test).
(DOCX)

S5 Table Expression of 17 deregulated metabolic genes in female patients. (DOCX)

S6 Table The combination model of risk metabolic genes on patient survival. (DOCX)

S7 Table Area under the curve of 34 risk metabolic genes in male patients. (DOCX)

S8 Table Area under the curve of 15 risk metabolic genes in female patients. (DOCX)

S9 Table Validation of risk metabolic genes in male and female patients in GSE72094 dataset. (DOCX)

S10 Table Validation of risk metabolic genes in male and female patients in GSE68465 dataset. (DOCX)

S11 Table The combination model of risk metabolic genes on patient survival in two validation datasets. (DOCX)
