# Supplementary material for: Transcriptome analysis reveals gender-specific differences in overall metabolic response of male and female patients in lung adenocarcinoma
Source: PLoS One. 2020 Apr 1;15(4):e0230796. doi: 10.1371/journal.pone.0230796 (PMC7112214; doi:10.1371/journal.pone.0230796)
Supplement: S1 Table — (DOCX) [file pone.0230796.s002.docx]

**Supplementary Table 1.** Commonly up-regulated metabolic genes in male and female.

| **Gene** | **Male (tumor vs. adjacent)** | | **Female (tumor vs. adjacent)** | |
| --- | --- | --- | --- | --- |
|  | **logFC** | **FDR** | **logFC** | **FDR** |
| A4GNT | 1.30 | 1.13E-13 | 1.10 | 4.76E-15 |
| AASDHPPT | 1.27 | 4.33E-05 | 1.17 | 2.31E-06 |
| ABCC2 | 1.44 | 2.37E-15 | 1.25 | 4.45E-19 |
| ABCD1 | 2.47 | 8.70E-10 | 2.13 | 1.61E-12 |
| ACLY | 1.19 | 5.86E-07 | 1.28 | 5.29E-12 |
| ALDOB | 1.75 | 6.43E-15 | 1.76 | 7.76E-25 |
| ASAH1 | 1.31 | 1.16E-11 | 1.15 | 2.07E-16 |
| ATP6V1B2 | 1.28 | 2.72E-11 | 1.15 | 5.18E-13 |
| B4GALNT3 | 2.02 | 6.36E-10 | 1.26 | 2.69E-07 |
| CES1 | 1.51 | 9.61E-06 | 1.31 | 2.11E-06 |
| CHKB | 1.30 | 1.84E-18 | 1.19 | 1.17E-23 |
| CLK1 | 1.69 | 2.51E-17 | 1.62 | 7.53E-28 |
| CTBP1 | 1.31 | 8.86E-18 | 1.15 | 3.43E-21 |
| CTSW | 1.96 | 7.80E-11 | 2.30 | 5.39E-25 |
| CYP3A43 | 1.31 | 5.23E-12 | 1.15 | 5.53E-15 |
| DUSP10 | 1.53 | 9.33E-08 | 1.50 | 4.77E-11 |
| ENOPH1 | 1.16 | 6.97E-17 | 1.04 | 8.54E-21 |
| ENPP1 | 1.49 | 6.83E-17 | 1.08 | 4.11E-15 |
| FASN | 2.16 | 6.26E-20 | 1.96 | 2.68E-26 |
| FKBP9 | 1.17 | 6.35E-16 | 1.12 | 1.19E-19 |
| GALNT3 | 1.29 | 8.38E-16 | 1.27 | 1.29E-19 |
| GGT1 | 1.21 | 3.54E-10 | 1.00 | 1.92E-10 |
| GGTLC1 | 1.06 | 6.61E-06 | 1.00 | 8.07E-09 |
| GLA | 2.24 | 2.99E-13 | 2.24 | 1.60E-19 |
| GMPS | 1.38 | 1.66E-14 | 1.21 | 3.61E-16 |
| GPIHBP1 | 1.42 | 7.57E-08 | 1.17 | 7.93E-09 |
| GYG2 | 1.79 | 4.03E-05 | 1.67 | 7.21E-08 |
| HDAC3 | 1.39 | 2.04E-07 | 1.38 | 5.43E-13 |
| HS6ST2 | 4.60 | 3.37E-19 | 4.13 | 1.02E-26 |
| LGMN | 1.43 | 7.70E-07 | 1.61 | 1.58E-11 |
| LYZL1 | 2.28 | 1.38E-18 | 1.96 | 3.72E-20 |
| MARK3 | 1.06 | 7.45E-19 | 1.01 | 2.00E-26 |
| MXD3 | 1.39 | 8.62E-09 | 1.48 | 2.35E-15 |
| MIB2 | 1.47 | 5.53E-06 | 1.16 | 1.68E-07 |
| NDUFA8 | 1.46 | 4.70E-08 | 1.35 | 8.00E-12 |
| NEK11 | 1.44 | 9.33E-13 | 1.37 | 1.94E-19 |
| NEURL1 | 1.31 | 1.83E-04 | 1.29 | 6.63E-07 |
| NT5C1B-RDH14 | 1.75 | 4.78E-16 | 1.54 | 3.77E-20 |
| PANK2 | 1.26 | 1.14E-12 | 1.07 | 6.53E-14 |
| PDE4B | 1.08 | 4.34E-09 | 1.04 | 8.06E-14 |
| PDP1 | 1.97 | 2.26E-16 | 1.69 | 1.52E-19 |
| PFAS | 2.15 | 8.94E-17 | 1.83 | 4.92E-19 |
| PLCB1 | 2.64 | 6.33E-14 | 2.70 | 2.54E-22 |
| PLCL1 | 1.04 | 5.01E-11 | 1.16 | 2.52E-17 |
| PLIN2 | 1.21 | 5.69E-08 | 1.01 | 1.00E-08 |
| PNPLA8 | 1.59 | 1.01E-14 | 1.48 | 2.33E-21 |
| POLR2D | 1.29 | 2.08E-12 | 1.31 | 4.54E-17 |
| POLR2E | 1.76 | 1.86E-08 | 1.96 | 4.56E-18 |
| PPP2R2B | 2.00 | 5.16E-18 | 1.88 | 9.06E-25 |
| PRKD1 | 1.11 | 1.01E-13 | 1.03 | 1.54E-20 |
| PTP4A3 | 1.42 | 2.30E-07 | 1.21 | 1.67E-12 |
| PTPN21 | 1.14 | 7.39E-07 | 1.01 | 3.05E-08 |
| RNF149 | 1.47 | 8.78E-09 | 1.25 | 8.48E-12 |
| RPIA | 2.17 | 2.33E-18 | 1.90 | 6.59E-25 |
| RPS6KA5 | 2.32 | 1.34E-25 | 1.97 | 2.95E-28 |
| SC5D | 1.38 | 2.92E-23 | 1.19 | 5.15E-25 |
| SIAH2 | 1.48 | 9.61E-05 | 1.39 | 6.73E-07 |
| SLC1A7 | 1.14 | 1.08E-12 | 1.08 | 8.85E-17 |
| SLC27A3 | 1.13 | 6.00E-09 | 1.18 | 5.05E-15 |
| SLC30A7 | 1.17 | 9.54E-13 | 1.08 | 1.54E-15 |
| SLC43A1 | 1.43 | 1.18E-14 | 1.30 | 6.02E-17 |
| SLC6A19 | 1.31 | 1.50E-04 | 1.24 | 5.51E-06 |
| SLC6A3 | 1.27 | 7.62E-07 | 1.14 | 2.24E-09 |
| SLC9A3 | 1.38 | 1.43E-12 | 1.14 | 1.04E-13 |
| SLCO1B3 | 1.18 | 1.56E-04 | 1.12 | 4.67E-06 |
| SORD | 1.31 | 1.51E-11 | 1.25 | 7.34E-14 |
| ST3GAL5 | 1.48 | 1.53E-06 | 1.38 | 9.06E-09 |
| TARSL2 | 1.48 | 8.59E-05 | 1.24 | 7.73E-06 |
| TBK1 | 1.27 | 1.05E-19 | 1.03 | 5.83E-22 |
| TNFRSF21 | 2.47 | 1.52E-11 | 2.41 | 5.32E-19 |
| TNS2 | 1.69 | 3.67E-17 | 1.49 | 3.69E-25 |
| TPP1 | 1.47 | 3.72E-13 | 1.18 | 3.01E-14 |
| UBE4B | 1.89 | 8.77E-06 | 1.70 | 1.12E-07 |
