# Supplementary material for: Transcriptome analysis reveals gender-specific differences in overall metabolic response of male and female patients in lung adenocarcinoma
Source: PLoS One. 2020 Apr 1;15(4):e0230796. doi: 10.1371/journal.pone.0230796 (PMC7112214; doi:10.1371/journal.pone.0230796)
Supplement: S2 Table — (DOCX) [file pone.0230796.s004.docx]

**Supplementary Table 2.** Commonly down-regulated metabolic genes in male and female.

| **Gene** | **Male (tumor vs. adjacent)** | | **Female (tumor vs. adjacent)** | |
| --- | --- | --- | --- | --- |
|  | **logFC** | **FDR** | **logFC** | **FDR** |
| ACAA2 | -1.72 | 2.07E-06 | -1.25 | 1.40E-05 |
| ACAD11 | -1.41 | 2.13E-12 | -1.14 | 6.53E-14 |
| ACO1 | -4.85 | 6.18E-25 | -4.37 | 1.23E-30 |
| ADH6 | -1.44 | 8.36E-24 | -1.50 | 1.46E-31 |
| AGPAT5 | -1.46 | 3.94E-19 | -1.42 | 5.01E-26 |
| AKR1A1 | -2.43 | 1.65E-23 | -2.29 | 8.25E-32 |
| ALPPL2 | -1.26 | 3.54E-10 | -1.15 | 2.75E-15 |
| AOC2 | -1.58 | 3.49E-11 | -1.38 | 1.33E-12 |
| APOC2 | -1.24 | 3.09E-04 | -1.36 | 1.23E-06 |
| ATP1B2 | -2.79 | 3.38E-20 | -2.67 | 2.16E-30 |
| ATP5G3 | -1.67 | 3.41E-21 | -1.89 | 5.08E-33 |
| B3GALT4 | -1.39 | 7.52E-12 | -1.31 | 5.79E-19 |
| BCO1 | -1.37 | 1.27E-12 | -1.23 | 1.96E-16 |
| BPGM | -2.14 | 9.17E-19 | -1.92 | 1.70E-21 |
| BRAF | -3.07 | 2.93E-04 | -3.27 | 1.72E-07 |
| CAMKK1 | -2.37 | 1.32E-19 | -2.21 | 1.08E-24 |
| CARM1 | -2.13 | 2.34E-26 | -1.93 | 1.90E-29 |
| CES5A | -2.26 | 2.16E-15 | -1.88 | 1.14E-17 |
| CHPF | -1.35 | 7.63E-19 | -1.43 | 2.31E-28 |
| CHST12 | -1.44 | 4.10E-15 | -1.18 | 1.83E-16 |
| CHSY1 | -1.38 | 1.95E-10 | -1.31 | 2.30E-16 |
| CMA1 | -1.84 | 5.96E-15 | -1.53 | 1.28E-13 |
| CSNK1A1L | -2.52 | 6.43E-35 | -2.69 | 5.00E-46 |
| CUBN | -1.18 | 2.98E-13 | -1.37 | 4.77E-24 |
| CYP11A1 | -1.01 | 5.01E-07 | -1.02 | 1.20E-09 |
| CYP26A1 | -1.87 | 1.93E-11 | -1.76 | 1.89E-16 |
| CYP26B1 | -1.12 | 4.41E-04 | -1.55 | 1.73E-10 |
| CYP2D6 | -2.09 | 9.60E-14 | -1.77 | 7.48E-14 |
| DEGS1 | -2.73 | 4.39E-38 | -2.57 | 1.18E-46 |
| DHRS3 | -1.08 | 5.39E-10 | -1.02 | 9.08E-16 |
| DNMT3B | -1.80 | 2.44E-15 | -1.58 | 2.26E-18 |
| DNMT3L | -1.10 | 1.51E-11 | -1.08 | 8.43E-18 |
| DSTYK | -3.42 | 2.64E-16 | -2.84 | 9.86E-14 |
| DUSP15 | -2.49 | 8.07E-26 | -2.32 | 5.85E-28 |
| DYRK2 | -2.20 | 1.13E-15 | -1.81 | 3.66E-19 |
| ETFB | -1.44 | 1.38E-22 | -1.08 | 9.06E-17 |
| EXT1 | -1.21 | 9.31E-09 | -1.21 | 4.51E-16 |
| FABP3 | -2.25 | 3.56E-21 | -2.10 | 1.12E-22 |
| FAM65A | -1.30 | 8.35E-12 | -1.34 | 6.54E-18 |
| FKBP1B | -2.39 | 9.01E-13 | -1.64 | 1.56E-08 |
| FMO5 | -1.16 | 8.52E-20 | -1.02 | 8.74E-25 |
| FNTB | -1.34 | 1.75E-42 | -1.41 | 1.89E-55 |
| GALC | -1.50 | 1.25E-22 | -1.52 | 1.05E-32 |
| GALM | -1.12 | 9.40E-16 | -1.03 | 7.42E-19 |
| GAPDH | -2.34 | 7.96E-16 | -1.56 | 3.63E-10 |
| GBE1 | -1.67 | 4.93E-14 | -1.49 | 7.17E-16 |
| GFPT2 | -1.45 | 7.87E-09 | -1.18 | 5.39E-10 |
| GLTP | -1.55 | 1.05E-29 | -1.76 | 4.03E-54 |
| GLUL | -1.02 | 3.64E-12 | -1.12 | 2.90E-22 |
| GRK2 | -1.46 | 2.06E-22 | -1.07 | 4.89E-18 |
| GSR | -1.51 | 4.83E-07 | -1.09 | 5.00E-05 |
| GSTA2 | -1.13 | 8.53E-05 | -1.23 | 1.74E-07 |
| HARS | -1.84 | 3.85E-14 | -1.24 | 1.31E-10 |
| HIPK2 | -1.46 | 1.52E-20 | -1.55 | 1.02E-34 |
| HMGCS2 | -2.21 | 7.59E-26 | -1.66 | 4.54E-17 |
| HS3ST2 | -1.29 | 1.80E-18 | -1.34 | 1.44E-21 |
| HS3ST4 | -1.10 | 6.40E-14 | -1.08 | 4.09E-22 |
| HS3ST6 | -2.42 | 2.33E-07 | -2.58 | 2.75E-14 |
| HSD11B2 | -2.20 | 8.34E-21 | -2.02 | 8.32E-27 |
| HSD17B6 | -1.86 | 1.68E-22 | -1.67 | 1.04E-26 |
| ITPK1 | -2.42 | 6.96E-28 | -2.49 | 2.33E-41 |
| KAT2A | -1.24 | 2.37E-17 | -1.18 | 4.96E-21 |
| KAT2B | -2.04 | 1.93E-12 | -1.91 | 1.23E-16 |
| LIMK2 | -1.68 | 9.68E-21 | -1.49 | 8.32E-25 |
| LSS | -1.31 | 2.75E-04 | -1.13 | 1.29E-05 |
| MANBA | -1.60 | 1.18E-13 | -1.30 | 7.85E-13 |
| MID1 | -1.05 | 1.52E-12 | -1.07 | 7.56E-20 |
| MTMR6 | -2.09 | 1.09E-10 | -1.85 | 1.48E-11 |
| MTR | -1.86 | 5.63E-15 | -1.64 | 2.65E-17 |
| MYLK3 | -1.54 | 6.58E-10 | -1.54 | 1.73E-18 |
| NANS | -1.78 | 1.29E-18 | -1.55 | 1.53E-22 |
| NDUFAB1 | -1.29 | 1.05E-18 | -1.47 | 2.30E-33 |
| NEK1 | -1.11 | 1.02E-09 | -1.01 | 2.19E-11 |
| NMT1 | -1.69 | 4.22E-10 | -1.51 | 1.40E-12 |
| NSDHL | -1.20 | 1.50E-09 | -1.23 | 1.21E-20 |
| NUDT10 | -1.97 | 2.60E-34 | -1.96 | 2.73E-44 |
| PAPD7 | -2.31 | 1.56E-16 | -2.32 | 1.58E-24 |
| PAPOLG | -1.68 | 2.77E-15 | -1.30 | 2.94E-13 |
| PDE1C | -1.74 | 8.58E-17 | -1.51 | 2.03E-17 |
| PDE9A | -1.45 | 3.50E-11 | -1.52 | 5.86E-18 |
| PDIA3 | -1.68 | 1.18E-14 | -1.31 | 5.26E-14 |
| PGLYRP1 | -1.72 | 8.10E-33 | -1.58 | 2.75E-43 |
| PLA2G2A | -1.15 | 9.45E-07 | -1.19 | 5.50E-11 |
| PLA2G4A | -1.52 | 1.92E-11 | -1.58 | 9.40E-20 |
| PLIN1 | -1.09 | 3.13E-08 | -1.22 | 3.68E-16 |
| PLPP2 | -1.94 | 5.66E-27 | -1.86 | 3.13E-34 |
| PPP2R3A | -1.46 | 8.14E-19 | -1.25 | 3.67E-20 |
| PRKAR2A | -2.38 | 2.24E-18 | -2.11 | 4.18E-25 |
| PRPF19 | -1.74 | 1.76E-13 | -1.45 | 4.95E-12 |
| PSKH2 | -1.56 | 4.23E-11 | -1.69 | 2.99E-21 |
| PTPN1 | -1.70 | 3.92E-14 | -1.44 | 3.76E-12 |
| PTPN12 | -1.22 | 4.68E-13 | -1.05 | 1.88E-14 |
| PTPN22 | -2.07 | 1.82E-30 | -2.22 | 3.08E-40 |
| PXDNL | -1.91 | 4.71E-08 | -1.72 | 5.67E-12 |
| QDPR | -1.75 | 9.70E-15 | -1.80 | 3.06E-23 |
| RIOK1 | -1.18 | 5.10E-16 | -1.21 | 2.02E-26 |
| RIPK4 | -3.63 | 6.66E-11 | -3.52 | 1.77E-15 |
| RRM1 | -1.12 | 1.70E-11 | -1.09 | 3.19E-18 |
| SIK2 | -1.24 | 1.29E-07 | -1.38 | 2.62E-18 |
| SLC10A2 | -1.70 | 1.52E-16 | -1.78 | 1.36E-22 |
| SLC15A4 | -1.62 | 5.49E-09 | -1.95 | 5.59E-20 |
| SLC26A4 | -1.39 | 1.18E-14 | -1.20 | 1.11E-14 |
| SLC29A3 | -1.29 | 3.29E-12 | -1.34 | 8.20E-20 |
| SLC34A1 | -1.26 | 2.66E-24 | -1.16 | 3.10E-30 |
| SLC35B4 | -1.44 | 1.50E-11 | -1.42 | 8.69E-17 |
| SLC37A4 | -1.06 | 3.36E-04 | -1.08 | 5.53E-06 |
| SLC5A9 | -2.68 | 1.33E-11 | -2.31 | 2.20E-11 |
| SLC9A5 | -1.07 | 6.89E-05 | -1.21 | 7.25E-09 |
| SOAT1 | -1.34 | 8.95E-11 | -1.13 | 2.13E-12 |
| SPHK1 | -1.84 | 1.52E-22 | -1.98 | 3.51E-32 |
| SRMS | -1.56 | 8.53E-15 | -1.26 | 5.42E-14 |
| ST3GAL4 | -1.12 | 1.52E-16 | -1.28 | 8.46E-28 |
| ST8SIA5 | -2.01 | 1.25E-27 | -2.23 | 8.70E-50 |
| STARD3 | -1.82 | 1.35E-18 | -1.18 | 7.68E-12 |
| TAOK2 | -8.11 | 2.04E-21 | -7.39 | 1.57E-26 |
| TP53RK | -3.32 | 9.33E-20 | -2.98 | 1.76E-23 |
| TRIT1 | -3.83 | 1.12E-35 | -3.84 | 1.08E-48 |
| UBE2L6 | -1.49 | 4.06E-11 | -1.27 | 9.08E-12 |
| UGT2B15 | -1.12 | 3.61E-08 | -1.26 | 1.53E-15 |
| USP13 | -1.64 | 1.68E-30 | -1.59 | 1.57E-38 |
| USP17L30 | -1.26 | 3.48E-16 | -1.05 | 7.27E-18 |
| USP8 | -1.20 | 5.18E-16 | -1.37 | 1.46E-28 |
| XPO7 | -2.00 | 7.73E-20 | -1.67 | 3.87E-21 |
