# Supplementary material for: Transcriptome analysis reveals gender-specific differences in overall metabolic response of male and female patients in lung adenocarcinoma
Source: PLoS One. 2020 Apr 1;15(4):e0230796. doi: 10.1371/journal.pone.0230796 (PMC7112214; doi:10.1371/journal.pone.0230796)
Supplement: S3 Fig — (DOCX) [file pone.0230796.s005.docx]

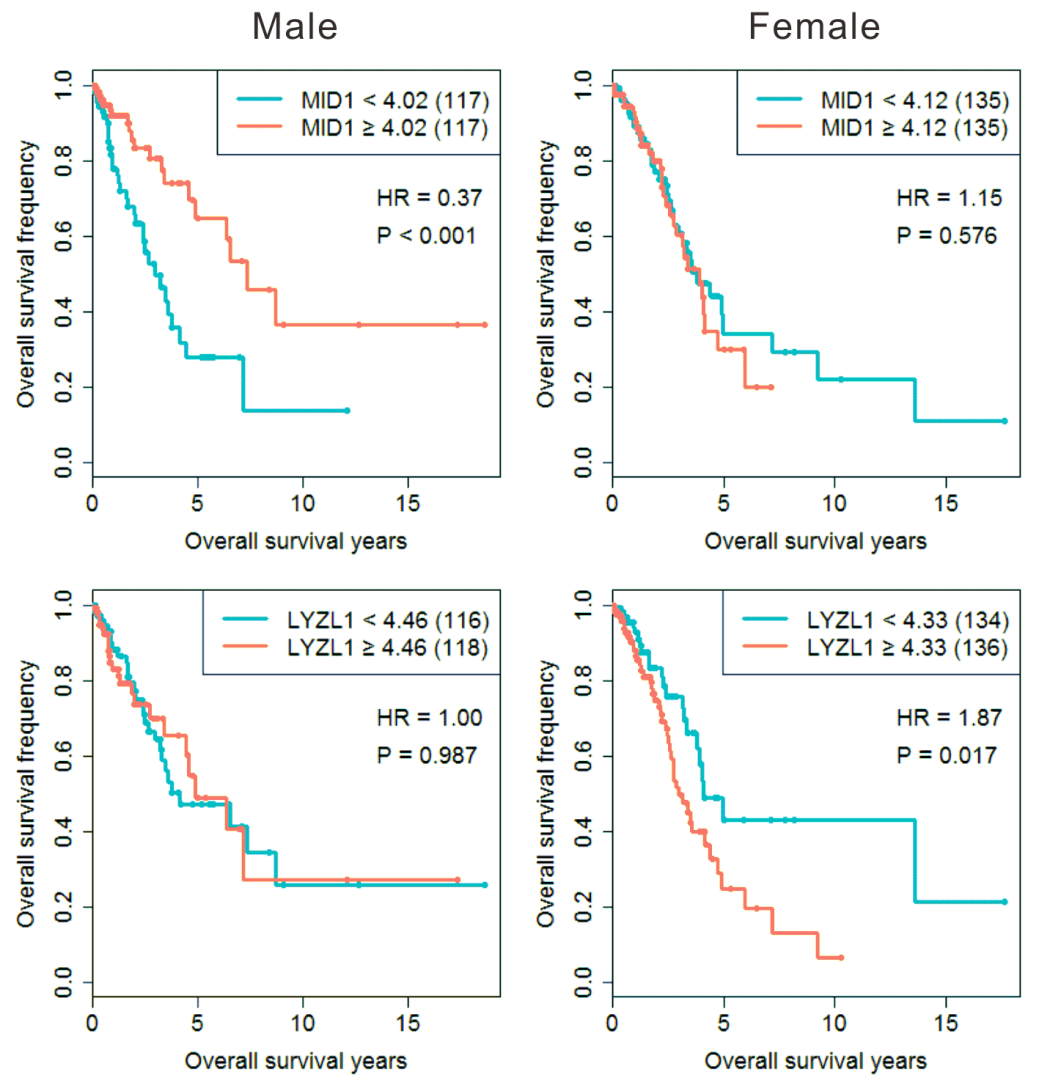


**Supplementary Figure 3.** Example of different effect of risk metabolic genes on prognosis in male and female patients.
