# Supplementary material for: Transcriptome analysis reveals gender-specific differences in overall metabolic response of male and female patients in lung adenocarcinoma
Source: PLoS One. 2020 Apr 1;15(4):e0230796. doi: 10.1371/journal.pone.0230796 (PMC7112214; doi:10.1371/journal.pone.0230796)
Supplement: S3 Table — (DOCX) [file pone.0230796.s006.docx]

**Supplementary Table 3.** Effect of 37 deregulated metabolic genes on male prognosis.

| **Name** | **HR (95% CI)** | **P value** | **FDR** |
| --- | --- | --- | --- |
| MID1 | 0.37 (0.21-0.64) | < 0.001 | 0.232 |
| A4GNT | 2.07 (1.18-3.62) | 0.011 | 0.394 |
| AASDHPPT | 2.25 (1.25-4.03) | 0.007 | 0.394 |
| AKR1C3 | 0.51 (0.30-0.88) | 0.016 | 0.394 |
| ASAH1 | 2.00 (1.15-3.48) | 0.014 | 0.394 |
| DSTYK | 0.49 (0.28-0.86) | 0.013 | 0.394 |
| ENPP1 | 2.08 (1.19-3.64) | 0.010 | 0.394 |
| GMPS | 2.08 (1.18-3.66) | 0.011 | 0.394 |
| HMGCS2 | 0.51 (0.29-0.89) | 0.018 | 0.394 |
| MAN2A1 | 1.98 (1.13-3.45) | 0.017 | 0.394 |
| NEK11 | 2.05 (1.18-3.57) | 0.011 | 0.394 |
| PDE1C | 0.48 (0.27-0.85) | 0.012 | 0.394 |
| PIK3C2A | 0.50 (0.29-0.88) | 0.015 | 0.394 |
| PTPN11 | 1.95 (1.13-3.38) | 0.017 | 0.394 |
| RIPK4 | 0.52 (0.30-0.90) | 0.019 | 0.394 |
| SLC29A3 | 2.02 (1.17-3.49) | 0.012 | 0.394 |
| STARD3 | 0.49 (0.28-0.85) | 0.012 | 0.394 |
| LSS | 0.52 (0.30-0.90) | 0.021 | 0.395 |
| ACLY | 1.91 (1.10-3.31) | 0.022 | 0.403 |
| CA13 | 1.92 (1.09-3.37) | 0.023 | 0.405 |
| HARS | 0.52 (0.30-0.92) | 0.024 | 0.405 |
| APOC2 | 0.55 (0.32-0.96) | 0.034 | 0.424 |
| NT5C1B-RDH14 | 1.82 (1.05-3.15) | 0.032 | 0.424 |
| PPP2R2B | 1.81 (1.05-3.12) | 0.033 | 0.424 |
| PTPN22 | 0.55 (0.31-0.95) | 0.033 | 0.424 |
| SLC27A3 | 0.54 (0.31-0.95) | 0.032 | 0.424 |
| SLC35B4 | 0.54 (0.31-0.94) | 0.030 | 0.424 |
| PTPN1 | 0.56 (0.33-0.96) | 0.036 | 0.429 |
| EXT1 | 0.56 (0.32-0.97) | 0.037 | 0.435 |
| FABP3 | 0.55 (0.32-0.97) | 0.038 | 0.435 |
| HS3ST2 | 0.55 (0.31-0.97) | 0.038 | 0.435 |
| NIT2 | 1.76 (1.02-3.03) | 0.042 | 0.444 |
| PLCB1 | 0.56 (0.33-0.98) | 0.041 | 0.444 |
| PRKACA | 0.56 (0.32-0.98) | 0.042 | 0.444 |
| SLC9A3 | 1.76 (1.02-3.04) | 0.043 | 0.444 |
| TAOK2 | 0.57 (0.33-0.99) | 0.047 | 0.457 |
| UBE2I | 0.57 (0.33-0.99) | 0.048 | 0.457 |
