# Supplementary material for: Transcriptome analysis reveals gender-specific differences in overall metabolic response of male and female patients in lung adenocarcinoma
Source: PLoS One. 2020 Apr 1;15(4):e0230796. doi: 10.1371/journal.pone.0230796 (PMC7112214; doi:10.1371/journal.pone.0230796)
Supplement: S4 Fig — The red box represents the metabolic subsystem that is upregulated, and the blue box represents the metabolic subsystem that is downregulated. The yellow circle indicates that the metabolic subsystem is significantly enriched. (DOCX) [file pone.0230796.s007.docx]

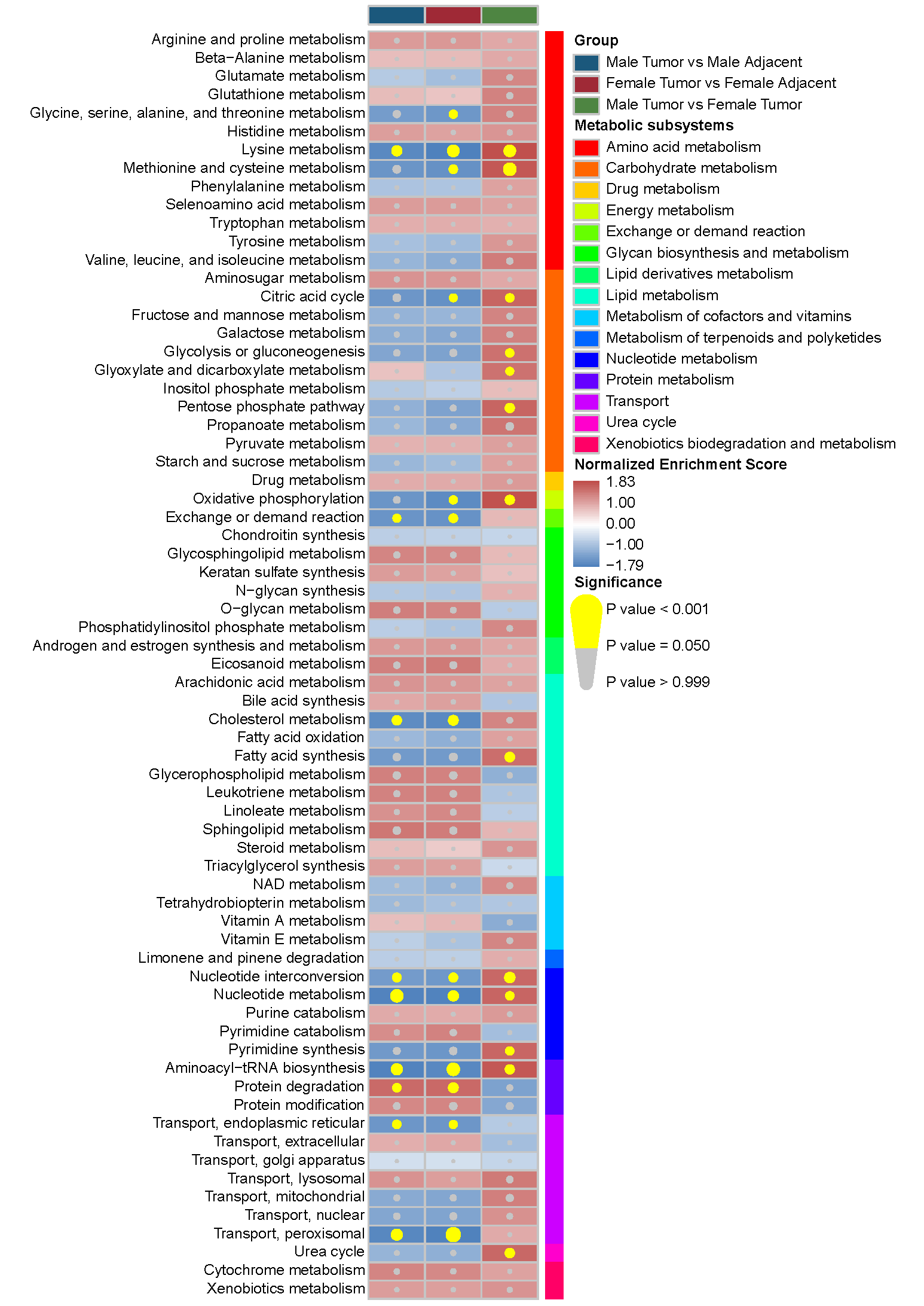


**Supplementary Figure 4.** Gene set enrichment analysis results of enriched metabolic subsystems in validation dataset (GSE68465). The red box represents the metabolic subsystem that is upregulated, and the blue box represents the metabolic subsystem that is downregulated. The yellow circle indicates that the metabolic subsystem is significantly enriched.
