# Supplementary material for: Transcriptome analysis reveals gender-specific differences in overall metabolic response of male and female patients in lung adenocarcinoma
Source: PLoS One. 2020 Apr 1;15(4):e0230796. doi: 10.1371/journal.pone.0230796 (PMC7112214; doi:10.1371/journal.pone.0230796)
Supplement: S4 Table — (DOCX) [file pone.0230796.s008.docx]

**Supplementary Table 4.** Effect of 17 deregulated metabolic genes on female prognosis.

| **Name** | **HR (95% CI)** | **P value** | **FDR** |
| --- | --- | --- | --- |
| NEK11 | 2.25 (1.34-3.76) | 0.002 | 0.492 |
| EXT1 | 0.46 (0.27-0.79) | 0.005 | 0.563 |
| SLCO1B3 | 2.01 (1.21-3.34) | 0.007 | 0.563 |
| ASAH1 | 1.89 (1.13-3.18) | 0.016 | 0.734 |
| LYZL1 | 1.87 (1.12-3.11) | 0.016 | 0.734 |
| RPS6KA5 | 0.54 (0.33-0.89) | 0.016 | 0.734 |
| ITPK1 | 0.53 (0.31-0.89) | 0.017 | 0.760 |
| HS6ST2 | 1.76 (1.08-2.86) | 0.024 | 0.801 |
| SLC43A1 | 1.76 (1.06-2.93) | 0.028 | 0.801 |
| SLC9A3 | 1.83 (1.09-3.08) | 0.023 | 0.801 |
| ABCC2 | 1.66 (1.01-2.74) | 0.047 | 0.834 |
| CARM1 | 0.58 (0.34-0.98) | 0.041 | 0.834 |
| CYP3A43 | 1.68 (1.02-2.77) | 0.041 | 0.834 |
| HDAC3 | 0.60 (0.36-0.98) | 0.043 | 0.834 |
| ST3GAL4 | 0.59 (0.35-0.97) | 0.039 | 0.834 |
| TP53RK | 0.59 (0.36-0.99) | 0.045 | 0.834 |
| TPP1 | 1.68 (1.01-2.77) | 0.044 | 0.834 |
