# Supplementary material for: Transcriptome analysis reveals gender-specific differences in overall metabolic response of male and female patients in lung adenocarcinoma
Source: PLoS One. 2020 Apr 1;15(4):e0230796. doi: 10.1371/journal.pone.0230796 (PMC7112214; doi:10.1371/journal.pone.0230796)
Supplement: S5 Fig — (A) The CCK-8 assay results showing the proliferation ability of A549 and XWLC-05 cells. Cells were transfected with overexpression ex-TAOK2 plasmid or blank vector (exCtrl).(B) The invasion assay results showing the invasion ability of A549 and XWLC-05 cells. The results were from three independent experiments. The cell number in each group was normalized to the control. Cells were transfected with overexpression ex-TAOK2 plasmid or blank vector (exCtrl).(C) The wound-healing assay results showing the migration ability of A549 and XWLC-05 cells. Cells were transfected with overexpression ex-TAOK2 or blank vector (exCtrl) and photos were taken in 40x field of vision. Images were taken in 40x field of vision.(*P < 0.05,**P < 0.01,***P < 0.001, Student’s t-test). (DOCX) [file pone.0230796.s009.docx]

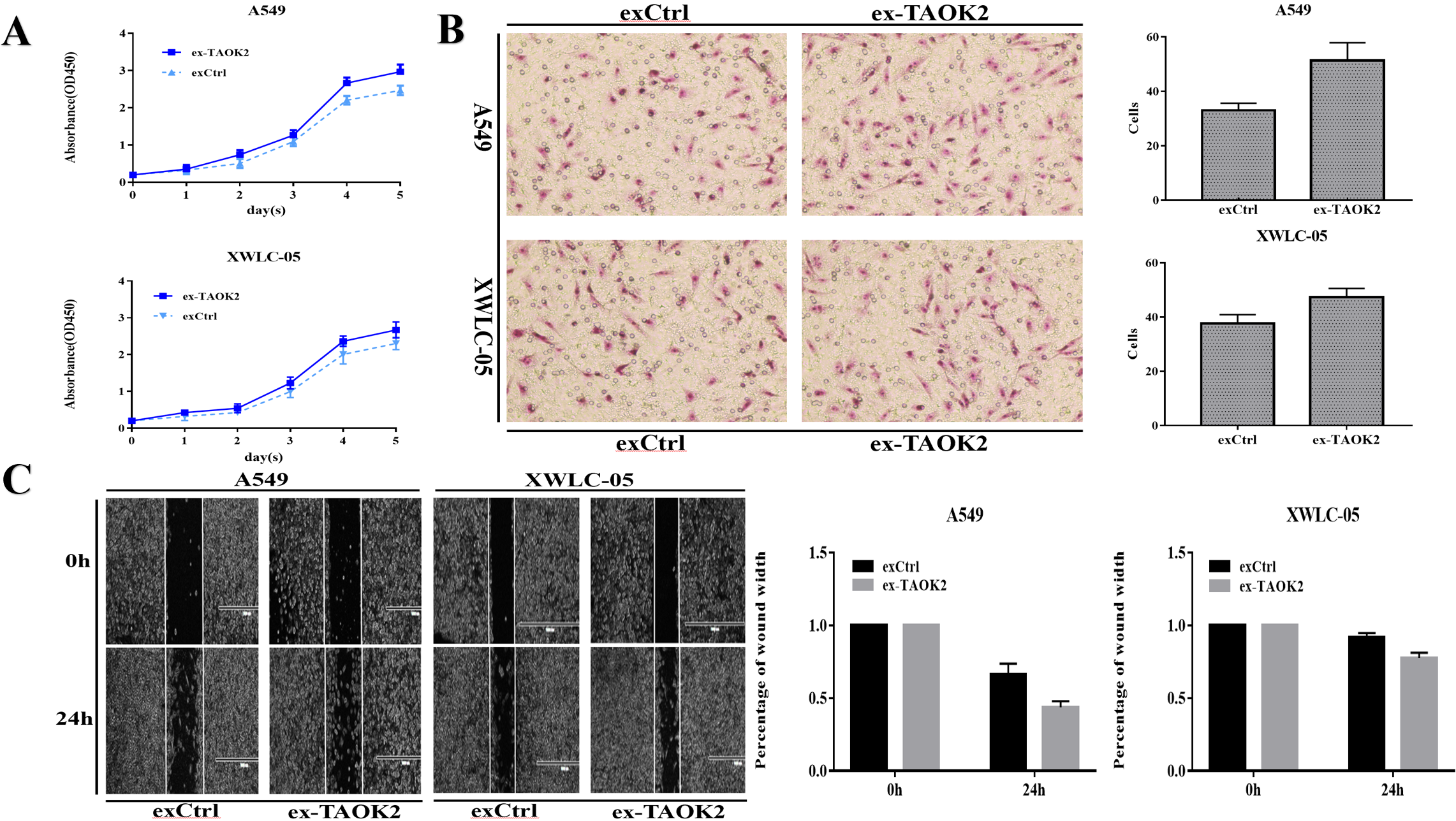


**Supplementary Figure 5.** Effect of overexpression of TAOK2 on the proliferation, migration, and invasion of lung adenocarcinoma cancer cells in vitro. (A) The CCK-8 assay results showing the proliferation ability of A549 and XWLC-05 cells. Cells were transfected with overexpression ex-TAOK2 plasmid or blank vector (exCtrl).(B) The invasion assay results showing the invasion ability of A549 and XWLC-05 cells. The results were from three independent experiments. The cell number in each group was normalized to the control. Cells were transfected with overexpression ex-TAOK2 plasmid or blank vector (exCtrl).(C) The wound-healing assay results showing the migration ability of A549 and XWLC-05 cells. Cells were transfected with overexpression ex-TAOK2 or blank vector (exCtrl) and photos were taken in 40x ﬁeld of vision. Images were taken in 40x ﬁeld of vision.(*P < 0.05,**P < 0.01,***P < 0.001, Student’s t-test).
