# Supplementary material for: Transcriptome analysis reveals gender-specific differences in overall metabolic response of male and female patients in lung adenocarcinoma
Source: PLoS One. 2020 Apr 1;15(4):e0230796. doi: 10.1371/journal.pone.0230796 (PMC7112214; doi:10.1371/journal.pone.0230796)
Supplement: S5 Table — (DOCX) [file pone.0230796.s010.docx]

**Supplementary Table 5.** Expression of 17 deregulated metabolic genes in female patients.

| **Name** | **Whole cohort** | | **Premenopausal**  **(age < 50, n = 18)** | | **Menopause**  **(age ≥ 50, n = 248)** | |
| --- | --- | --- | --- | --- | --- | --- |
|  | **LogFC** | **FDR** | **LogFC** | **FDR** | **LogFC** | **FDR** |
| NEK11 | 1.37 | 1.94E-19 | 1.82 | 1.50E-20 | 1.39 | 1.04E-31 |
| EXT1 | -1.21 | 4.51E-16 | -1.09 | 1.60E-11 | -1.18 | 4.10E-24 |
| SLCO1B3 | 1.12 | 4.67E-06 | 0.97 | 1.22E-05 | 1.22 | 3.77E-11 |
| ASAH1 | 1.15 | 2.07E-16 | 1.52 | 1.22E-18 | 1.09 | 2.58E-25 |
| LYZL1 | 1.96 | 3.72E-20 | 2.89 | 4.78E-24 | 2.04 | 3.61E-33 |
| RPS6KA5 | 1.97 | 2.95E-28 | 2.33 | 2.03E-15 | 2.08 | 3.00E-44 |
| ITPK1 | -2.49 | 2.33E-41 | -2.64 | 7.40E-13 | -2.43 | 3.82E-53 |
| HS6ST2 | 4.13 | 1.02E-26 | 4.11 | 9.66E-15 | 4.29 | 2.16E-41 |
| SLC43A1 | 1.30 | 6.02E-17 | 1.82 | 9.18E-22 | 1.26 | 3.22E-26 |
| SLC9A3 | 1.14 | 1.04E-13 | 1.68 | 1.94E-21 | 1.10 | 2.83E-21 |
| ABCC2 | 1.25 | 4.45E-19 | 1.69 | 1.42E-18 | 1.22 | 2.38E-30 |
| CARM1 | -1.93 | 1.90E-29 | -2.34 | 1.80E-22 | -1.95 | 3.27E-44 |
| CYP3A43 | 1.15 | 5.53E-15 | 1.67 | 4.08E-16 | 1.10 | 1.52E-24 |
| HDAC3 | 1.38 | 5.43E-13 | 1.23 | 2.26E-10 | 1.32 | 6.80E-19 |
| ST3GAL4 | -1.28 | 8.46E-28 | -0.80 | 0.001465 | -1.23 | 2.33E-48 |
| TP53RK | -2.98 | 1.76E-23 | -3.81 | 3.21E-28 | -3.03 | 8.92E-37 |
| TPP1 | 1.18 | 3.01E-14 | 1.33 | 5.10E-17 | 1.17 | 2.72E-22 |
