# Supplementary material for: Transcriptome analysis reveals gender-specific differences in overall metabolic response of male and female patients in lung adenocarcinoma
Source: PLoS One. 2020 Apr 1;15(4):e0230796. doi: 10.1371/journal.pone.0230796 (PMC7112214; doi:10.1371/journal.pone.0230796)
Supplement: S6 Table — (DOCX) [file pone.0230796.s011.docx]

**Supplementary Table 6.** The combination model of risk metabolic genes on patient survival.

| **Male** | | | | **Female** | | | |
| --- | --- | --- | --- | --- | --- | --- | --- |
| **Gene** | **nloglik^1^** | **AIC^2^** | **Selected^3^** | **Gene** | **nloglik^1^** | **AIC^2^** | **Selected^3^** |
| NEK11 | 817.36 | 1636.71 | * | TP53RK | 958.62 | 1919.24 | * |
| HS3ST2 | 815.45 | 1634.90 | * | TPP1 | 955.46 | 1914.92 | * |
| ACLY | 815.21 | 1636.43 | * | ST3GAL4 | 954.62 | 1915.23 | * |
| HARS | 813.32 | 1634.63 | * | LYZL1 | 951.58 | 1911.16 | * |
| SLC35B4 | 810.44 | 1630.89 | * | ITPK1 | 950.66 | 1911.31 | * |
| MID1 | 809.82 | 1631.65 |  | ASAH1 | 949.91 | 1911.82 | * |
| STARD3 | 809.32 | 1632.63 |  | CYP3A43 | 948.01 | 1910.02 | * |
| EXT1 | 809.14 | 1634.28 |  | SLC9A3 | 947.83 | 1911.65 |  |
| PRKACA | 809.01 | 1636.02 |  | CARM1 | 947.7 | 1913.40 |  |
| PPP2R2B | 808.9 | 1637.79 |  | SLC43A1 | 947.47 | 1914.95 |  |

^1^ nlogliks: negative log-likelihoods

^2^ AIC: Akaike's An Information Criterion

^3^ The genes in the optimal model with the smallest AIC are marked with asterisks.
