# Supplementary material for: Transcriptome analysis reveals gender-specific differences in overall metabolic response of male and female patients in lung adenocarcinoma
Source: PLoS One. 2020 Apr 1;15(4):e0230796. doi: 10.1371/journal.pone.0230796 (PMC7112214; doi:10.1371/journal.pone.0230796)
Supplement: S7 Table — (DOCX) [file pone.0230796.s012.docx]

**Supplementary Table 7.** Area under the curve of 34 risk metabolic genes in male patients.

| **Name** | **Whole cohort (n = 237)** | | | **Stage I and II (n = 180)** | | |
| --- | --- | --- | --- | --- | --- | --- |
|  | **AUC** | **95% CI** | **Power^1^** | **AUC** | **95% CI** | **Power^1^** |
| TAOK2 | 1.000 | 0.999-1.000 | 1.000 | 1.000 | 1.000-1.000 | 1.000 |
| A4GNT | 0.988 | 0.978-0.988 | 1.000 | 0.990 | 0.979-0.990 | 1.000 |
| PTPN22 | 0.987 | 0.975-0.987 | 1.000 | 0.984 | 0.970-0.984 | 1.000 |
| MAN2A1 | 0.986 | 0.973-0.986 | 1.000 | 0.987 | 0.975-0.987 | 1.000 |
| NT5C1B-RDH14 | 0.981 | 0.966-0.981 | 1.000 | 0.983 | 0.967-0.983 | 1.000 |
| GMPS | 0.978 | 0.962-0.978 | 1.000 | 0.979 | 0.962-0.979 | 1.000 |
| ASAH1 | 0.972 | 0.954-0.972 | 1.000 | 0.976 | 0.957-0.976 | 1.000 |
| HMGCS2 | 0.972 | 0.953-0.972 | 1.000 | 0.972 | 0.951-0.972 | 1.000 |
| NIT2 | 0.971 | 0.949-0.971 | 1.000 | 0.973 | 0.951-0.973 | 1.000 |
| PPP2R2B | 0.971 | 0.952-0.971 | 1.000 | 0.974 | 0.955-0.974 | 1.000 |
| DSTYK | 0.966 | 0.944-0.966 | 1.000 | 0.962 | 0.936-0.962 | 1.000 |
| ENPP1 | 0.964 | 0.942-0.964 | 1.000 | 0.967 | 0.944-0.967 | 1.000 |
| FABP3 | 0.964 | 0.941-0.964 | 1.000 | 0.969 | 0.946-0.969 | 1.000 |
| NEK11 | 0.963 | 0.940-0.963 | 1.000 | 0.964 | 0.939-0.964 | 1.000 |
| SLC9A3 | 0.961 | 0.938-0.961 | 1.000 | 0.961 | 0.937-0.961 | 1.000 |
| PDE1C | 0.954 | 0.929-0.954 | 1.000 | 0.947 | 0.917-0.947 | 1.000 |
| HARS | 0.953 | 0.929-0.953 | 1.000 | 0.947 | 0.918-0.947 | 1.000 |
| RIPK4 | 0.943 | 0.915-0.943 | 1.000 | 0.946 | 0.916-0.946 | 1.000 |
| STARD3 | 0.942 | 0.906-0.942 | 1.000 | 0.936 | 0.895-0.936 | 1.000 |
| PTPN1 | 0.941 | 0.909-0.941 | 1.000 | 0.938 | 0.904-0.938 | 1.000 |
| MID1 | 0.932 | 0.892-0.932 | 1.000 | 0.939 | 0.898-0.939 | 1.000 |
| CA13 | 0.925 | 0.885-0.925 | 1.000 | 0.926 | 0.885-0.926 | 1.000 |
| HS3ST2 | 0.922 | 0.879-0.922 | 1.000 | 0.921 | 0.875-0.921 | 1.000 |
| PTPN11 | 0.915 | 0.879-0.915 | 1.000 | 0.917 | 0.878-0.917 | 1.000 |
| ACLY | 0.884 | 0.838-0.884 | 1.000 | 0.894 | 0.844-0.894 | 1.000 |
| EXT1 | 0.879 | 0.828-0.879 | 1.000 | 0.883 | 0.829-0.883 | 1.000 |
| PRKACA | 0.875 | 0.829-0.875 | 1.000 | 0.854 | 0.800-0.854 | 1.000 |
| UBE2I | 0.858 | 0.806-0.858 | 1.000 | 0.839 | 0.779-0.839 | 1.000 |
| SLC35B4 | 0.853 | 0.762-0.853 | 1.000 | 0.847 | 0.755-0.847 | 1.000 |
| AKR1C3 | 0.811 | 0.741-0.811 | 1.000 | 0.795 | 0.719-0.795 | 0.998 |
| AASDHPPT | 0.803 | 0.742-0.803 | 0.999 | 0.812 | 0.748-0.812 | 0.999 |
| LSS | 0.770 | 0.714-0.770 | 0.994 | 0.759 | 0.695-0.759 | 0.988 |
| APOC2 | 0.766 | 0.690-0.766 | 0.992 | 0.757 | 0.675-0.757 | 0.987 |
| PIK3C2A | 0.726 | 0.646-0.726 | 0.958 | 0.684 | 0.596-0.684 | 0.838 |

^1^ Expected power of the test (1 - probability of type II error).
