# Supplementary material for: Transcriptome analysis reveals gender-specific differences in overall metabolic response of male and female patients in lung adenocarcinoma
Source: PLoS One. 2020 Apr 1;15(4):e0230796. doi: 10.1371/journal.pone.0230796 (PMC7112214; doi:10.1371/journal.pone.0230796)
Supplement: S8 Table — (DOCX) [file pone.0230796.s013.docx]

**Supplementary Table 8.** Area under the curve of 15 risk metabolic genes in female patients.

| **Name** | **Whole cohort (n = 276)** | | | **Stage I and II (n = 215)** | | |
| --- | --- | --- | --- | --- | --- | --- |
|  | **AUC** | **95% CI** | **Power^1^** | **AUC** | **95% CI** | **Power^1^** |
| ASAH1 | 0.985 | 0.968-0.985 | 1.000 | 0.983 | 0.963-0.983 | 1.000 |
| CYP3A43 | 0.980 | 0.964-0.980 | 1.000 | 0.976 | 0.957-0.976 | 1.000 |
| ABCC2 | 0.973 | 0.954-0.973 | 1.000 | 0.970 | 0.949-0.970 | 1.000 |
| SLC43A1 | 0.971 | 0.944-0.971 | 1.000 | 0.967 | 0.937-0.967 | 1.000 |
| CARM1 | 0.963 | 0.937-0.963 | 1.000 | 0.961 | 0.934-0.961 | 1.000 |
| TP53RK | 0.962 | 0.943-0.962 | 1.000 | 0.963 | 0.941-0.963 | 1.000 |
| HS6ST2 | 0.960 | 0.937-0.960 | 1.000 | 0.960 | 0.935-0.960 | 1.000 |
| TPP1 | 0.954 | 0.928-0.954 | 1.000 | 0.952 | 0.924-0.952 | 1.000 |
| NEK11 | 0.952 | 0.926-0.952 | 1.000 | 0.944 | 0.914-0.944 | 1.000 |
| LYZL1 | 0.950 | 0.925-0.950 | 1.000 | 0.942 | 0.913-0.942 | 1.000 |
| ST3GAL4 | 0.948 | 0.916-0.948 | 1.000 | 0.956 | 0.923-0.956 | 1.000 |
| SLC9A3 | 0.944 | 0.917-0.944 | 1.000 | 0.934 | 0.902-0.934 | 1.000 |
| ITPK1 | 0.941 | 0.890-0.941 | 1.000 | 0.941 | 0.889-0.941 | 1.000 |
| EXT1 | 0.920 | 0.885-0.920 | 1.000 | 0.914 | 0.876-0.914 | 1.000 |
| SLCO1B3 | 0.733 | 0.679-0.733 | 0.996 | 0.733 | 0.673-0.733 | 0.995 |

^1^ Expected power of the test (1 - probability of type II error).
