# Supplementary material for: Transcriptome analysis reveals gender-specific differences in overall metabolic response of male and female patients in lung adenocarcinoma
Source: PLoS One. 2020 Apr 1;15(4):e0230796. doi: 10.1371/journal.pone.0230796 (PMC7112214; doi:10.1371/journal.pone.0230796)
Supplement: S10 Table — (DOCX) [file pone.0230796.s015.docx]

**Supplementary Table 10.** Validation of risk metabolic genes in male and female patients in GSE68465 dataset.

| **Risk metabolic genes in male** | **Trend in differential expression^1^** | **Trend in survival analysis^2^** | **Risk metabolic genes in female** | **Trend in differential expression^1^** | **Trend in survival analysis^2^** |
| --- | --- | --- | --- | --- | --- |
| A4GNT | Y | N | ABCC2 | N | Y |
| AASDHPPT | N | Y | ASAH1 | Y | N |
| ACLY | N | Y | CYP3A43 | Y | N |
| ASAH1 | Y | N | NEK11 | Y | Y |
| ENPP1 | N | Y | SLC43A1 | N | N |
| GMPS | N | Y | SLC9A3 | Y | N |
| MAN2A1 | N | Y | SLCO1B3 | Y | Y |
| NEK11 | Y | N | TPP1 | Y | N |
| NIT2 | N | Y | CARM1 | Y | Y |
| PPP2R2B | Y | Y | EXT1 | N | N |
| PTPN11 | N | Y | ITPK1 | N | Y |
| SLC9A3 | Y | N | ST3GAL4 | Y | N |
| AKR1C3 | N | N |  |  |  |
| APOC2 | Y | N |  |  |  |
| DSTYK | Y | Y |  |  |  |
| EXT1 | N | N |  |  |  |
| FABP3 | N | N |  |  |  |
| HARS | Y | N |  |  |  |
| HMGCS2 | N | Y |  |  |  |
| HS3ST2 | N | Y |  |  |  |
| LSS | Y | Y |  |  |  |
| MID1 | N | N |  |  |  |
| PDE1C | Y | N |  |  |  |
| PIK3C2A | Y | N |  |  |  |
| PRKACA | Y | Y |  |  |  |
| PTPN1 | N | Y |  |  |  |
| PTPN22 | N | N |  |  |  |
| RIPK4 | Y | Y |  |  |  |
| STARD3 | N | N |  |  |  |
| TAOK2 | Y | Y |  |  |  |
| UBE2I | Y | Y |  |  |  |

^1^ Y: The gene has the same expression trend in TCGA data and this dataset, N: The gene has a different expression trend in TCGA data and this dataset.

^2^ Y: The gene has the same prognosis trend in TCGA data and this dataset, N: The gene has a different prognosis trend in TCGA data and this dataset.
