# Supplementary material for: Transcriptome analysis reveals gender-specific differences in overall metabolic response of male and female patients in lung adenocarcinoma
Source: PLoS One. 2020 Apr 1;15(4):e0230796. doi: 10.1371/journal.pone.0230796 (PMC7112214; doi:10.1371/journal.pone.0230796)
Supplement: S11 Table — (DOCX) [file pone.0230796.s016.docx]

**Supplementary Table 11.** The combination model of risk metabolic genes on patient survival in two validation datasets.

| **GSE68465** | | | | | | | |
| --- | --- | --- | --- | --- | --- | --- | --- |
| **Male** | | | | **Female** | | | |
| **Gene** | **nloglik^1^** | **AIC^2^** | **Selected^3^** | **Gene** | **nloglik^1^** | **AIC^2^** | **Selected^3^** |
| ASAH1 | 338.38 | 678.76 | * | ST3GAL4 | 483.05 | 968.10 | * |
| PPP2R2B | 334.67 | 673.35 | * | ITPK1 | 481.41 | 966.81 | * |
| MAN2A1 | 330.34 | 666.69 | * | ABCC2 | 480.94 | 967.89 |  |
| MID1 | 327.69 | 663.39 | * | SLC9A3 | 480.9 | 969.80 |  |
| A4GNT | 327.54 | 665.08 | * | SLCO1B3 | 480.29 | 970.57 |  |
| SLC9A3 | 327.53 | 667.05 | * | CARM1 | 480.19 | 972.38 |  |
| AASDHPPT | 327.20 | 668.40 | * | TPP1 | 480.06 | 974.12 |  |
| LSS | 326.55 | 669.09 | * | ASAH1 | 480.06 | 976.12 |  |
| PRKACA | 323.89 | 665.77 | * | CYP3A43 | 479.82 | 977.65 |  |
| ACLY | 319.35 | 658.70 | * | EXT1 | 479.79 | 979.58 |  |
| **GSE72094** | | | | | | | |
| **Male** | | | | **Female** | | | |
| **Gene** | **nloglik^1^** | **AIC^2^** | **Selected^3^** | **Gene** | **nloglik^1^** | **AIC^2^** | **Selected^3^** |
| AASDHPPT | 459.86 | 921.72 | * | ASAH1 | 721.29 | 1444.59 | * |
| ASAH1 | 458.63 | 921.27 | * | SLC9A3 | 721.05 | 1446.10 | * |
| HARS | 457.20 | 920.40 | * | SLCO1B3 | 717.63 | 1441.27 | * |
| PDE1C | 454.78 | 917.56 | * | CYP3A43 | 714.18 | 1436.35 | * |
| CA13 | 454.34 | 918.68 | * | ITPK1 | 712.89 | 1435.77 | * |
| PIK3C2A | 453.92 | 919.83 | * | TP53RK | 711.76 | 1435.53 | * |
| PTPN11 | 450.77 | 915.54 | * | HS6ST2 | 711.56 | 1437.11 |  |
| MID1 | 450.07 | 916.13 |  | NEK11 | 711.50 | 1439.00 |  |
| ENPP1 | 450.06 | 918.13 |  | ST3GAL4 | 711.49 | 1440.98 |  |
| GMPS | 448.97 | 917.93 |  | EXT1 | 711.15 | 1442.30 |  |

^1^ nlogliks: negative log-likelihoods

^2^ AIC: Akaike's An Information Criterion

^3^ The genes in the optimal model with the smallest AIC are marked with asterisks.
